# Exploring causal correlations between inflammatory cytokines and knee osteoarthritis: a two-sample Mendelian randomization

**DOI:** 10.3389/fimmu.2024.1362012

**Published:** 2024-04-18

**Authors:** Jiayu Zhang, Kexuan Li, Xiuyue Qiu

**Affiliations:** Nursing School, Zhejiang Chinese Medical University, Hangzhou, Zhejiang, China

**Keywords:** knee osteoarthritis, inflammatory cytokines, Mendelian randomization, disease etiology, genetic variation

## Abstract

**Objectives:**

Knee osteoarthritis (KOA) and certain inflammatory cytokines (such as interleukin 1 [IL-1] and tumor necrosis factor alpha [TNF-a]) are related; however, the causal relationship remains unclear. Here, we aimed to assess the causal relationship between 41 inflammatory cytokines and KOA using Mendelian randomization (MR).

**Methods:**

Two-sample bidirectional MR was performed using genetic variation data for 41 inflammatory cytokines that were obtained from European Genome-Wide Association Study (GWAS) data (n=8293). KOA-related genetic association data were also obtained from European GWAS data (n=40,3124). Inverse variance weighting (IVW), MR, heterogeneity, sensitivity, and multiple validation analyses were performed.

**Results:**

Granulocyte colony-stimulating factor (G-CSF) or colony-stimulating factor 3 (CSF-3) levels were negatively associated with the risk of developing KOA (OR: 0.93, 95%CI:0.89–0.99, *P*=0.015). Additionally, macrophage inflammatory protein-1 alpha (MIP-1A/CCL3) was a consequence of KOA (OR: 0.72, 95%CI:0.54–0.97, *P*=0.032). No causal relationship was evident between other inflammatory cytokines and KOA development.

**Conclusion:**

This study suggests that certain inflammatory cytokines may be associated with KOA etiology. G-CSF exerts an upstream influence on KOA development, whereas MIP-1A (CCL-3) acts as a downstream factor.

## 1 Introduction

Knee osteoarthritis (KOA) is a chronic degenerative disease that profoundly affects the joints and associated tissues (1). The pathological features of KOA encompass degenerative changes in the articular cartilage, secondary osteophyte formation, subchondral bone sclerosis, and synovial inflammation (2, 3). Epidemiological surveys have revealed that the prevalence of osteoarthritis (OA) is as high as 29% in the middle-aged population (4). Furthermore, the prevalence of primary KOA is as high as 46.3% in people aged > 40 years in China (5). The number of patients with KOA increases with an aging population (6). The primary clinical manifestations of KOA include pain, limited mobility, and joint deformity (7), which can affect joint stability and may lead to disability in severe cases (8). Approximately 100 million people worldwide are disabled due to KOA. Moreover, KOA accounts for 2.2% of the global disease burden and is the fourth most disabling disease worldwide (9, 10). Beyond its physical toll, KOA further affects the mental well-being of afflicted individuals and profoundly diminishes their overall quality of life (11). Simultaneously, the economic ramifications impose a substantial burden on patients, their families, and society (12). The annual healthcare costs associated with arthritis exceed $ 300 billion (13). Given the staggering incidence and disability associated with KOA, identifying modifiable risk factors is imperative for developing strategies to control the disease and alleviate its societal burden.

Cytokines are small-molecule proteins with a wide range of biological activities that are synthesized and secreted by immune and certain non-immune cells in response to various stimuli (14). Cytokines have various functions, including immunomodulation, cell growth, and tissue repair (15). Cytokines can be categorized into interleukins, chemokines, and growth factors (16). The exact etiology and pathogenesis of KOA remain unclear. However, it is now generally accepted that inflammation is involved in the development of the disease (17), and it has been suggested that KOA is triggered by multiple complex factors mediated by inflammatory cytokines and their associated signaling pathways (18, 19). Inflammatory cytokines are closely associated with functional alterations in the synovium, cartilage, and subchondral bone and can impair the synthetic pathways required to repair the integrity of degenerating chondrocytes (20). The association between inflammation and KOA has been demonstrated in animal model studies (21). Furthermore, certain inflammatory cytokines, such as IL-1 and tumor necrosis factor (TNF-ɑ), can be detected during early lesions of KOA, a finding that suggests that inflammatory cytokines are associated with KOA (22–24). The expression levels of inflammatory cytokines in joint fluids influence KOA to some extent (25). However, relevant studies have mainly focused on the effects of IL-1 and TNF-a on KOA (26–28), and few studies regarding the association between other inflammatory cytokines and KOA exist. In addition, owing to the limitations of the traditional study design and the inability of the current study to completely exclude confounding factors, the causal relationship between inflammatory cytokines and KOA remains unclear and should be confirmed by additional studies.

Mendelian randomization (MR) uses genetic variants associated with modifiable exposures (or risk factors) to assess their possible causal relationship with outcomes (28–31). Single nucleotide polymorphisms (SNPs) are commonly used as IVs to assess potential causal associations with outcomes (32, 33). Because genetic variants are randomly assigned at the time of conception, potential sources of error are eliminated, and a clear causal chain is established (34). This minimizes potential bias due to confounding factors and reverse causation, thus increasing the reliability of the results (35). Current MR in KOA mostly focuses on MR between two diseases or influencing factors, such as OA with cardiovascular disease and osteoporosis (36, 37). However, there is a notable gap between the studies addressing the potential causal relationship between KOA and inflammatory cytokine levels. To address this gap, we aimed to assess the potential causal association between inflammatory cytokines and KOA using two-sample bidirectional MR analysis. We first extracted validated genetic IVs for 41 inflammatory cytokines from the genome-wide association study (GWAS) data and analyzed their association with KOA. The direction of causality was further explored using reverse exposure and outcomes. Our study findings potentially provide new strategies for KOA prevention.

## 2 Materials and methods

### 2.1 MR assumptions

**Figure 1:**
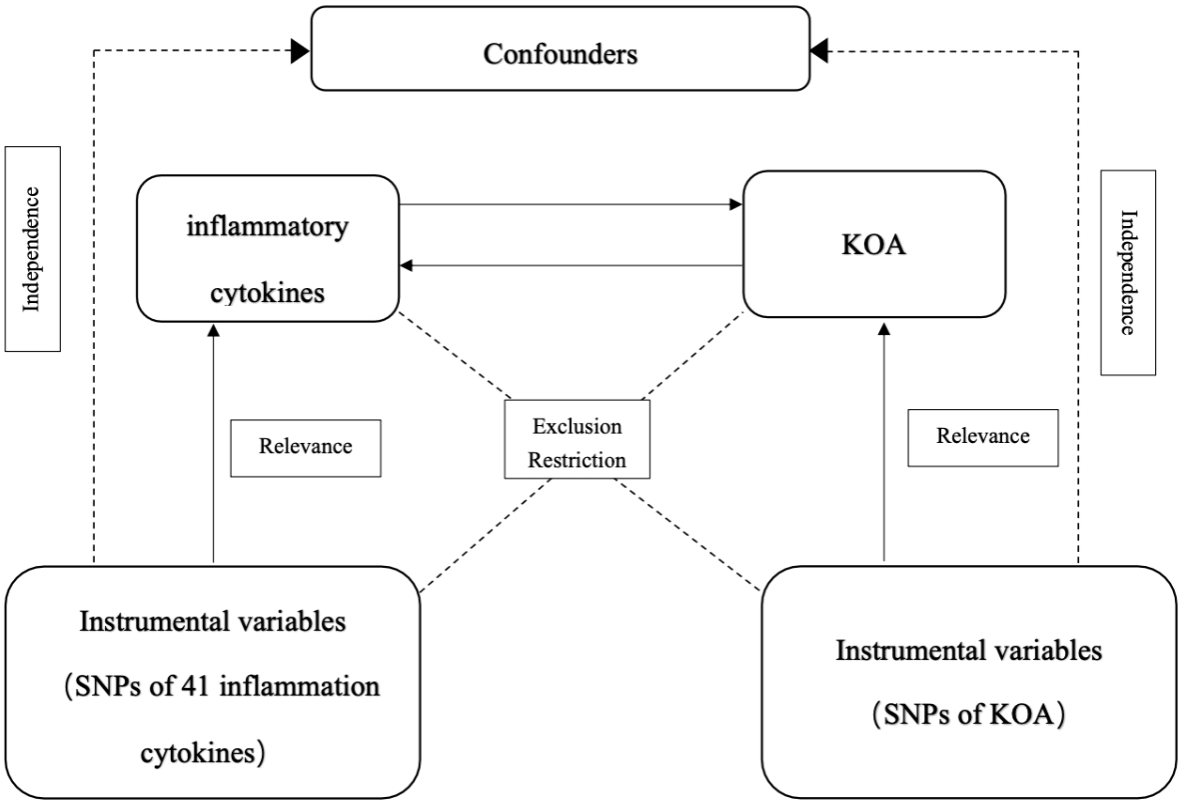
Schematic representation of the study design in this bidirectional Mendelian randomization (MR) analysis.

MR was used to analyze the relationship between inflammatory cytokines and KOA. **Figure 1** shows the two-sample bidirectional MR study design. There are three core assumptions of MR analysis: relevance, independence, and exclusion. (1) Relevance: the variables selected as genetic instruments are closely associated with exposures; (2) independence: genetic variation is not associated with confounding factors; and (3) exclusion restrictions: genetic variation affects outcomes only through exposures instead of other pathways (32).

### 2.2 Data source

The two datasets used in the MR analysis were derived from publicly available GWAS summary data. The KOA data for this study were obtained from the UK Biobank and Arthritis Research UK Osteoarthritis Genetics (arcOGEN). This study included 24,955 patients and 378,169 controls of European ancestry (38) (https://was.mrcieu.ac.uk/datasets/ebi-a-GCST007090/). The UK Biobank recruited participants aged 40–69 between 2006–2010. ArcOGEN is a collection of patients of European ancestry with KOA. Diagnosis of KOA was based on clinical signs of the disease and the need for arthroplasty or imaging evidence (Kelgren-Lawrence≥2) to determine the diagnosis. For 41 inflammatory cytokines, data were obtained from a meta-analysis combining the results of the Finnish Young Adult Cardiovascular Risk Study (YFS) and the FINRISK survey between 1980–2011, which included 8,293 Finnish individuals (39) (https://data.bris.ac.uk/data/dataset/3g3i5smgghp0s2uvm1doflkx9x). The mean age of the participants in the YFS an FINRISK studies were 37 and 60 years, respectively.

### 2.3 Instrumental variable selection

First, we set *P*<5×10^-8^ as the genome-wide significance threshold to select SNPs that were strongly associated with KOA and inflammatory cytokines. Because very few SNPs were identified for some of the cytokines upon exposure, a higher cutoff value (*P*<5×10^-6^) to ensure that there were enough SNPs for further MR analysis (40, 41). Secondly, to avoid linkage disequilibrium, instrumental variables were removed to ensure mutual independence of these instrumental variables (r2 = 0.001, kb=10,000). Third, the *F* statistic was used to determine the IV exposure correlations. The *F* value of the SNP was calculated to determine the presence of a weak IV bias. If *F*>10, the correlation is considered strong enough to avoid weak IV bias.

### 2.4 Statistical analysis

In this study, we explored the potential causal relationship between inflammatory cytokines and KOA using inflammatory cytokines and KOA as exposure and outcome variables, respectively. Analysis was performed using R4.3.2 software and the R package “Two sample MR”. Causality assessments were conducted using diverse methods including inverse variance weighting (IVW), MR-Egger, weighted median, weighted modal, and simple modal analyses. The IVW method served as the primary approach for the MR analysis, and Cochran’s Q value was computed for both the IVW and MR-Egger estimates to gauge heterogeneity. Horizontal pleiotropy was evaluated using the MR-Egger intercept test (42) and anomalous SNPs were identified using MR-PRESSO (43). The stability of the results was examined using the leave-one-out method, wherein each SNP was systematically excluded to assess its impact on supporting a causal association (44). In cases where IVW analysis yielded a significant result (*P*<0.05) and no evidence of horizontal pleiotropy or heterogeneity was found, the result could be considered positive, even if other methods did not yield a significant result, as long as the direction of the beta remained consistent (42). In cases where horizontal pleiotropy was present but no heterogeneity was detected, the MR-Egger method was used. In cases where heterogeneity was observed, but multidirectionality was not present, the analysis was performed using the IVW method.

## 3 Results

To ensure an adequate number of SNPs for further MR analysis, a significance threshold of *P*<5×10^-6^ was selected when screening for SNPs related to each inflammatory cytokine and KOA. The *F*-statistics for the SNPs of the 41 inflammatory cytokines were 20.77–782.26. The *F*-statistic for all instrumental variables was >10, indicating no weak instrumental variable bias (**Supplementary Tables 1-3**).

To determine the primary analytical tool, hypothesis checks were performed for all 41 inflammatory cytokines. The IVW method was used as the primary analytical method for all cytokines, except IL-1RA, for which there was no evidence of heterogeneity or weak interference. For IL-1RA, MR-Egger was chosen as the primary method and the reason for this was because the *P*-values of the Q-tests for both IVW and MR-Egger were <0.05 (*P*=0.032, *P*=0.040).

### 3.1 Influence of 41 inflammatory cytokines on KOA

**Figure 2:**
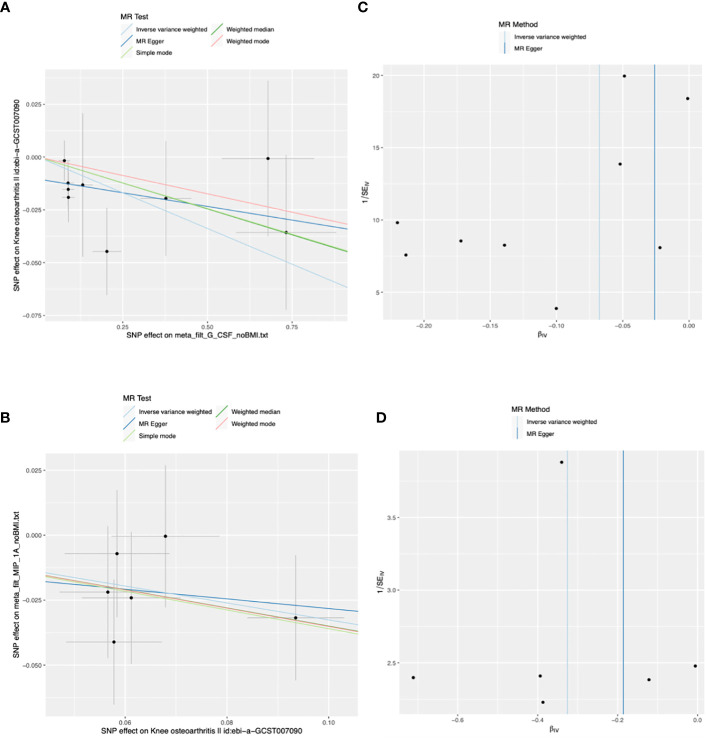
Scatter and funnel plots of Mendelian randomization (MR) analyses for granulocyte colony-stimulating factor (G-CSF) and macrophage inflammatory protein-1 alpha (MIP-1A) in patients with knee osteoarthritis (KOA). **(A)** The exposure is G-CSF and outcome is KOA. **(B)** The exposure is KOA and outcome is MIP-1A. **(C, D)** Funnel plots show the inverse variance weighted MR estimate of each cytokine single-nucleotide polymorphism (SNP) with KOA versus 1/standard error (1/SEIV).

The main results of the MR analysis using 41 inflammatory cytokines as exposures and KOA as the outcome are shown in **Tables 1** and **Supplementary Table 1**. The IVW results showed that genetically predicted elevated levels of Granulocyte colony-stimulating factor (G-CSF) were negatively correlated with the risk of developing KOA (OR:0.93, 95%CI:0.89–0.99, *P*=0.015). There was no evidence of potential horizontal pleiotropy (*P*=0.173) or heterogeneity (*P*=0.597) (**Supplementary Table 2**). MR-PRESSO analyses did not identify any SNP abnormalities in the G-CSF. The leave-one-out method demonstrates the stability of the results (**Supplementary Figure 1**). The beta values of the MR-Egger, simple model, weighted median, and weighted model analyses are consistent with the direction of the results. **Figures 2A, C** shows a scatter plot and funnel plot of the causal relationship between G-CSF and the occurrence of KOA. There is no evidence suggesting that other circulating inflammatory cytokines are associated with KOA.

**Table 1:** Causality of 41 circulating inflammatory cytokines on KOA.

N	EXPOSURES	nSNP	OR (95% CI)	*P*			
				IVW	Het	MR-EGGER	MR-PRESSO
1	B-NGF	4	1.03 (0.95,1.12)	0.503	0.286	0.634	0.39
2	CTACK	13	1.00 (0.96,1.03)	0.921	0.477	0.441	0.41
3	EOTAXIN	16	1.02 (0.97,1.08)	0.391	0.129	0.096	0.14
4	FGF-BASIC	7	0.99 (0.92,1.07)	0.775	0.612	0.505	0.75
5	G-CSF	9	0.93 (0.89,0.99)	**0.015**	0.597	0.763	0.68
6	GROA	11	1.00 (0.97,1.03)	0.840	0.467	0.706	0.55
7	HGF	9	0.98 (0.92,1.05)	0.643	0.602	0.736	0.67
8	IFN-G	12	0.98 (0.92,1.04)	0.508	0.346	0.311	0.43
9	IL-1B	3	0.95 (0.85,1.07)	0.416	0.171	0.142	/
10	IL-1RA	10	0.96 (0.89,1.04)	0.299	0.032	0.040	0.02
11	IL-2	8	0.98 (0.91,1.04)	0.472	0.064	0.105	0.14
12	IL-2RA	8	0.97 (0.93,1.02)	0.194	0.175	0.320	0.33
13	IL-4	14	0.98 (0.92,1.03)	0.417	0.291	0.278	0.47
14	IL-5	8	1.03 (0.98,1.09)	0.227	0.829	0.792	0.88
15	IL-6	11	1.01 (0.95,1.08)	0.649	0.404	0.342	0.36
16	IL-7	13	1.02 (0.98,1.06)	0.455	0.090	0.063	0.12
17	IL-8	8	0.98 (0.93,1.04)	0.521	0.071	0.167	0.15
18	IL-9	6	1.03 (0.97,1.09)	0.329	0.828	0.708	0.86
19	IL-10	15	1.03 (0.98,1.08)	0.294	0.182	0.243	0.23
20	IL-12-P70	14	1.03 (0.99,1.07)	0.163	0.308	0.244	0.37
21	IL-13	13	1.00 (0.97,1.04)	0.874	0.264	0.254	0.25
22	IL-16	9	1.01 (0.97,1.05)	0.728	0.119	0.144	0.19
23	IL-17	8	0.96 (0.90,1.02)	0.203	0.489	0.878	0.44
24	IL-18	13	1.01 (0.98,1.05)	0.473	0.096	0.097	0.15
25	IP-10	11	1.00 (0.95,1.04)	0.856	0.751	0.800	0.78
26	M-CSF	11	1.01 (0.98,1.05)	0.375	0.632	0.592	0.64
27	MCP-1-MCAF	14	0.97 (0.91,1.04)	0.399	0.054	0.051	0.08
28	MCP-3	6	1.01 (0.97,1.04)	0.786	0.586	0.465	0.61
29	MIF	10	1.01 (0.97,1.06)	0.580	0.685	0.621	0.68
30	MIG	13	0.98 (0.95,1.02)	0.403	0.744	0.880	0.79
31	MIP-1A	6	1.04 (0.97,1.11)	0.290	0.894	0.822	0.93
32	MIP-1B	20	1.02 (1.00,1.05)	0.108	0.466	0.402	0.56
33	PDGF-BB	14	0.97 (0.93,1.01)	0.151	0.727	0.658	0.82
34	RANTES	10	1.04 (0.99,1.09)	0.141	0.639	0.554	0.64
35	SCF	11	0.99 (0.93,1.06)	0.718	0.251	0.190	0.30
36	SCGF-B	18	1.01 (0.97,1.04)	0.753	0.224	0.187	0.31
37	SDF-1A	9	0.99 (0.93,1.05)	0.651	0.992	0.983	0.99
38	TNF-A	4	0.96 (0.91,1.02)	0.185	0.991	0.968	0.99
39	TNF-B	5	0.99 (0.95,1.03)	0.580	0.137	0.152	0.25
40	TRAIL	16	1.02 (0.99,1.05)	0.297	0.225	0.250	0.20
41	VEGF	15	1.03 (0.99,1.07)	0.123	0.076	0.054	0.16

CI, confidence interval; OR, odds ratio; SNP, single nucleotide polymorphism.

**Table 1** summarizes the results with 41 inflammatory cytokines as exposure and KOA as outcome.

### 3.2 Influence of KOA on 41 inflammatory cytokines

The main results of the MR analysis when KOA was used as the exposure and the 41 inflammatory cytokines were used as outcomes are shown in **Tables 2** and **Supplementary Table 4**. Six significant SNPs were extracted as instrumental variables for KOA, and the *F*-statistics for SNPs of KOA were 32.15–96.87(**Supplementary Tables 4-6**). As no heterogeneity or weak instruments were observed, the IVW method was used for the primary evaluation of SNP in KOA. The IVW results showed that genetically predicted increases in KOA occurrence were negatively associated with MIP-1A levels (OR: 0.72, 95%CI:0.54–0.97, *P*=0.032). MR-Egger analysis did not reveal any potential horizontal pleiotropy (*P*=0.862) or heterogeneity (*P*=0.880) of SNPs for exposure factors. MR-PRESSO analyses did not reveal outliers in the SNPs for the exposure factors (**Supplementary Table 5**). Leave-one-out analysis demonstrated the stability of the results (**Supplementary Figure 2**). The directions of the beta of the MR-Egger, simple model, weighted median, and weighted model analysis results were consistent. Scatter and funnel plots of the causality of MIP-1A in KOA are shown in **Figures 2B, D**. There is no evidence suggesting that KOA development is associated with other circulating inflammatory cytokines.

**Table 2:** Causality of KOA and 41 circulating inflammatory cytokines.

N	EXPOSURES	nSNP	Beta (95% CI)	*P*			
				IVW	Het	MR-EGGER	MR-PRESSO
1	B-NGF	6	0.77 (0.57,1.03)	0.08	0.567	0.432	0.61
2	CTACK	6	0.91 (0.66, 1.25)	0.55	0.319	0.355	0.32
3	EOTAXIN	6	1.07 (0.88,1.30)	0.51	0.810	0.760	0.85
4	FGF-BASIC	6	0.98 (0.80,1.20)	0.85	0.782	0.705	0.76
5	G-CSF	6	1.01 (0.83,1.24)	0.90	0.949	0.922	0.92
6	GROA	6	1.06 (0.79, 1.43)	0.71	0.523	0.771	0.52
7	HGF	6	1.01 (0.82,1.23)	0.96	0.381	0.260	0.44
8	IFN-G	6	1.08 (0.87,1.34)	0.49	0.333	0.270	0.39
9	IL-1B	6	1.01 (0.74,1.37)	0.95	0.991	0.990	1
10	IL-1RA	6	0.85 (0.63,1.14)	0.28	0.752	0.616	0.81
11	IL-2	6	0.91 (0.68,1.23)	0.54	0.663	0.522	0.74
12	IL-2RA	6	0.97 (0.66,1.43)	0.88	0.111	0.062	0.15
13	IL-4	6	1.16 (0.95,1.41)	0.15	0.720	0.581	0.68
14	IL-5	6	0.73 (0.49, 1.09)	0.12	0.133	0.095	0.2
15	IL-6	6	1.05 (0.87,1.28)	0.60	0.569	0.458	0.58
16	IL-7	6	0.79 (0.58,1.06)	0.12	0.609	0.464	0.68
17	IL-8	6	0.97 (0.72,1.31)	0.86	0.957	0.952	0.92
18	IL-9	6	0.98 (0.73,1.32)	0.90	0.882	0.798	0.9
19	IL-10	6	0.85 (0.69,1.04)	0.11	0.532	0.766	0.44
20	IL-12-P70	6	0.89 (0.73,1.08)	0.23	0.493	0.433	0.51
21	IL-13	6	0.79 (0.57,1.10)	0.17	0.274	0.189	0.32
22	IL-16	6	0.98 (0.73,1.32)	0.90	0.786	0.678	0.8
23	IL-17	3	1.05 (0.76, 1.45)	0.76	0.850	0.796	/
24	IL-18	6	1.33 (0.95,1.86)	0.10	0.259	0.213	0.34
25	IP-10	6	0.95 (0.71,1.26)	0.70	0.623	0.601	0.64
26	M-CSF	6	0.94 (0.64, 1.37)	0.75	0.330	0.234	0.41
27	MCP-1-MCAF	6	1.01 (0.80,1.28)	0.90	0.219	0.151	0.31
28	MCP-3	6	1.44 (0.85, 2.45)	0.18	0.481	0.344	0.56
29	MIF	6	0.93 (0.67,1.30)	0.68	0.285	0.193	0.38
30	MIG	6	0.89 (0.67,1.20)	0.45	0.994	0.994	1
31	MIP-1A	6	0.72 (0.54,0.97)	**0.03**	0.880	0.785	0.95
32	MIP-1B	6	1.06 (0.87,1.28)	0.59	0.539	0.596	0.51
33	PDGF-BB	6	1.05 (0.86,1.28)	0.63	0.682	0.971	0.58
34	RANTES	6	1.08 (0.76,1.52)	0.68	0.269	0.171	0.37
35	SCF	6	1.02 (0.82,1.27)	0.83	0.288	0.219	0.26
36	SCGF-B	6	0.83 (0.62,1.10)	0.20	0.796	0.926	0.76
37	SDF-1A	6	0.97 (0.79,1.19)	0.78	0.403	0.280	0.45
38	TNF-A	6	1.07 (0.80,1.45)	0.64	0.662	0.518	0.7
39	TNF-B	6	1.03 (0.66, 1.61)	0.90	0.947	0.926	0.97
40	TRAIL	6	0.97 (0.79,1.18)	0.74	0.659	0.535	0.66
41	VEGF	6	1.16 (0.91,1.48)	0.23	0.249	0.267	0.26

CI, confidence interval; SNP, single nucleotide polymorphism.

**Table 2** summarizes the results with KOA as the exposure and 41 inflammatory cytokines as the outcome.

## 4 Discussion

This study used a two-sample MR analysis to explore the potential causal relationship between inflammatory cytokines and KOA. First, we identified causal relationships between 41 inflammatory cytokines and KOA and found that lower levels of G-CSF were associated with a high risk of developing KOA, suggesting that G-CSF may be a potential upstream cause of KOA. In addition, when KOA is used as an exposure variable in MR, it may lead to lower MIP-1A levels via pathogenic pathways; therefore, MIP-1A may be located downstream of disease progression. In summary, our analysis suggests that G-CSF is involved in KOA pathogenesis. MIP-1A has a protective effect against KOA, as evidenced by the reduction in MIP-1A levels when KOA was considered an exposure factor.

Our findings suggested that reduced G-CSF levels were associated with an increased risk of KOA. G-CSF (also known as CSF-3) is a hematopoietic growth factor produced primarily by macrophages, vascular endothelial cells, fibroblasts, and bone marrow mesenchymal stromal cells (45). G-CSF is widely found in the body and the circulatory system (46) and is involved in chronic inflammatory autoimmune diseases such as rheumatoid arthritis (RA) (47). RA contributes directly to joint deterioration by mediating the release of a variety of cells, including macrophages and lymphocytes, as well as inflammatory factors: TNF-α, IL-1 and matrix metalloproteinases (48), which in turn manifests as joint deformities and dysfunction. This finding implies that G-CSF may have similar effects in patients with KOA. G-CSF primarily acts on hematopoietic progenitor cells to promote proliferation and differentiation, and stimulates granulocyte maturation and release (49–51). Reduced levels of G-CSF lead to decreased chemotaxis of granulocytes to damaged tissues (52), leading to decreased granulocyte numbers and activity (53, 54), thereby affecting immune cell function (55) and leading to reduced repair capacity of tissues, such as articular cartilage. Damage to articular cartilage and subchondral bone is an underlying pathological process of OA (56). G-CSF affects the survival and function of chondrocytes in the joint fluid (57), and reduced levels alter the state of these cells, affecting the stability of joint tissues (58), ultimately leading to KOA.

MIP-1A (also known as CCL3), belongs to the chemokine CC subfamily. MIP-1A is produced by a variety of cell types, including monocytes, fibroblasts, vascular endothelial cells, and smooth muscle cells (59), has chemotactic effects on monocytes, T cells, B cells (60, 61), and is involved in the pathogenesis of several inflammatory diseases such as RA and asthma (62–64). In patients with RA, MIP-1A triggers signaling pathways by binding to receptors on the surface of chondrocytes, leading to altered activation of intracellular molecules and inhibition of proteoglycan synthesis, ultimately affecting chondrocyte function (65, 66). In patients with RA, decreased MIP-1A expression correlates with disease severity, and there is a trend toward decreased MIP-1A expression with decreased macrophage infiltration (67). RA and KOA are both inflammatory diseases (68); therefore, it is hypothesized that MIP-1A may have a similar effect on KOA. A study on the synovial fluid also reported that MIP-1A levels decreased with OA progression (69).

Lowering MIP-1A levels may exert a protective effect against KOA through multiple pathways, including influencing immune cell activity, slowing inflammatory responses, and maintaining articular cartilage stability. First, lowering MIP-1A levels decreases immune cell activity (70). MIP-1A reduces the destructive effects of macrophages on joint tissues by attenuating their activation, thereby protecting joint structures, maintaining joint tissues (71), and slowing the progression of arthritis. Secondly, as an important chemokine (72), reduced levels of MIP-1A may lead to reduced immune cell chemotaxis in the affected joint region (73). This in turn slows the inflammatory response (74), thereby reducing inflammation and attenuating joint tissue degradation. Finally, MIP-1A affects chondrocyte function (75), leading to limited cellular synthesis of proteoglycans, which reduces proteoglycan synthesis and exacerbates articular cartilage damage (76). Therefore, a reduction in MIP-1A levels promotes proteoglycan synthesis, which reduces the risk of articular cartilage damage and protects joints.

Here, we found that MIP-1A levels were decreased in patients with KOA, whereas the study by Guo et al. (77) reported that MIP-1A levels were increased in patients with KOA, leading to a discrepancy, which may be due to the difference in the sample sizes of the studies, as well as the racial differences between the European and Asian populations. Therefore, future studies should explore the potential mechanism of the negative correlation between KOA and MIP-1A to reveal the specific role of MIP-1A in the pathogenesis of KOA, which will provide new perspectives on the treatment strategy of KOA and provides new ideas for discovering new ways to treat KOA.

The previous studies investigated the potential causal relationship between cytokines and OA, suggesting a correlation between CX3CL1, MCP4, and CCL25 and OA (78). Another study noted a causal relationship between the inflammatory cytokines MCSF and vascular endothelial growth factor (VEGF) and OA (79), but these results were not validated in our analysis. Potential explanations include differences in study focus, with previous research encompassing various types of OA, while our study specifically examined KOA. Variation in the selection of inflammatory cytokines across studies, each with distinct biological functions and expression patterns, may also contribute to differing results. Additionally, the heterogeneity of study populations could impact cytokine expression levels and patterns, influencing study outcomes. Notably, our study had a large sample size and assessed numerous cytokines. Furthermore, we identified previously unexplored causal relationships, such as the association between G-CSF and MIP-1A with KOA. These novel findings warrant further validation in future research to ascertain the potential of G-CSF and MIP-1A as biomarkers for KOA prevention and treatment.

This study has several limitations. First, our survey data were derived from two large-scale GWAS datasets, which make this study less statistically valid. However, owing to the lack of data, we were unable to perform subgroup analyses of the variables to refine the results of this study. Therefore, they must be validated in future large-sample clinical trials. Second, the GWAS data used in this study were derived from individuals of European origin, and there may be racial bias. Thus, the generalizability of our findings to other populations should be validated using local data. Third, a significance threshold with a *P*<5×10^-6^ was used in the exposed GWAS data because the number of genome-wide significant SNP at a threshold of *P*<5×10^-8^ was too small to support this study. Fourth, the scope of this study was limited to examining the correlation between the 41 inflammatory cytokines and KOA. Given the diversity of inflammatory cytokines, future studies should be extended to the latest 91 inflammatory cytokines to deepen our understanding of the causal relationships between inflammatory cytokines and KOA.

## 5 Conclusion

The results of this study suggest a causal relationship between inflammation and KOA. G-CSF (CSF-3) may be an upstream factor in KOA, whereas MIP-1A (CCL3) may be a downstream effect of KOA. However, further studies are required to determine whether these cytokines can be used to predict or prevent KOA.

## Author contributions

JZ: Writing – original draft. KL: Writing – review & editing. XQ: Writing – review & editing.
