# Supplementary material for: Exploring causal correlations between inflammatory cytokines and knee osteoarthritis: a two-sample Mendelian randomization
Source: Front Immunol. 2024 Apr 18;15:1362012. doi: 10.3389/fimmu.2024.1362012 (PMC11063282; doi:10.3389/fimmu.2024.1362012)
Supplement: Supplementary file 1 [file Table_1.docx]

**Table S1. MR estimates of forty-one inflammatory cytokines on KOA.**

| **N** | **EXPOSURES** | **nSNP** | **MR Egger** | | **Weighted median** | | **Inverse variance weighted** | | **Simple mode** | | **Weighted mode** | |
| --- | --- | --- | --- | --- | --- | --- | --- | --- | --- | --- | --- | --- |
|  |  |  | ***P*** | **OR (95%CI)** | ***P*** | **OR (95%CI)** | ***P*** | **OR (95%CI)** | ***P*** | **OR (95%CI)** | ***P*** | **OR (95%CI)** |
| 1 | B-NGF | 4 | 0.214 | 1.50 (0.96,2.30) | 0.386 | 1.04 (0.95,1.15) | 0.503 | 1.03 (0.95,1.12) | 0.397 | 1.07 (0.93,1.22) | 0.459 | 1.05 (0.94,1.18) |
| 2 | CTACK | 13 | 0.505 | 0.98 (0.92,1.04) | 0.379 | 1.02 (0.97,1.08) | 0.921 | 1.00 (0.96,1.03) | 0.508 | 1.03 (0.94,1.12) | 0.305 | 1.03 (0.97,1.10) |
| 3 | EOTAXIN | 16 | 0.653 | 1.03 (0.91,1.17) | 0.746 | 1.01 (0.95,1.08) | 0.391 | 1.02 (0.97,1.08) | 0.797 | 1.01 (0.92,1.12) | 0.808 | 1.01 (0.94,1.08) |
| 4 | FGF-BASIC | 7 | 0.857 | 1.01 (0.88,1.17) | 0.968 | 1.00 (0.90,1.11) | 0.775 | 0.99 (0.92,1.07) | 0.954 | 1.00 (0.86,1.15) | 0.968 | 1.00 (0.89,1.13) |
| 5 | G-CSF | 9 | 0.528 | 0.97 (0.90,1.05) | 0.205 | 0.95 (0.88,1.03) | 0.015 | 0.93 (0.89,0.99) | 0.381 | 0.95 (0.85,1.06) | 0.409 | 0.97 (0.90,1.04) |
| 6 | GROA | 11 | 0.152 | 1.05 (0.99,1.12) | 0.942 | 1.00 (0.97,1.04) | 0.840 | 1.00 (0.97,1.03) | 0.947 | 1.00 (0.94,1.06) | 0.720 | 1.01 (0.97,1.05) |
| 7 | HGF | 9 | 0.182 | 0.90 (0.78,1.04) | 0.360 | 0.96 (0.88,1.05) | 0.643 | 0.98 (0.92,1.05) | 0.525 | 0.96 (0.85,1.08) | 0.469 | 0.96 (0.86,1.07) |
| 8 | IFN-G | 12 | 0.360 | 0.94 (0.83,1.07) | 0.689 | 0.98 (0.91,1.07) | 0.508 | 0.98 (0.92,1.04) | 0.826 | 0.99 (0.88,1.11) | 0.740 | 0.98 (0.89,1.09) |
| 9 | IL-1B | 3 | 0.490 | 0.85 (0.63,1.15) | 0.831 | 0.99 (0.88,1.11) | 0.416 | 0.95 (0.85,1.07) | 0.926 | 1.01 (0.88,1.16) | 0.975 | 1.00 (0.87,1.15) |
| 10 | IL-1RA | 10 | 0.215 | 0.87 (0.71,1.07) | 0.045 | 0.92 (0.85,1.00) | 0.299 | 0.96 (0.89,1.04) | 0.153 | 0.89 (0.76,1.03) | 0.106 | 0.89 (0.78,1.01) |
| 11 | IL-2 | 8 | 0.189 | 0.91 (0.81,1.03) | 0.322 | 0.97 (0.90,1.04) | 0.472 | 0.98 (0.91,1.04) | 0.529 | 0.96 (0.86,1.08) | 0.322 | 0.96 (0.89,1.04) |
| 12 | IL-2RA | 8 | 0.783 | 1.01 (0.95,1.07) | 0.564 | 0.99 (0.94,1.03) | 0.194 | 0.97 (0.93,1.02) | 0.597 | 0.98 (0.89,1.07) | 0.514 | 0.98 (0.94,1.03) |
| 13 | IL-4 | 14 | 0.263 | 0.95 (0.86,1.04) | 0.619 | 0.98 (0.91,1.06) | 0.417 | 0.98 (0.92,1.03) | 0.695 | 1.02 (0.92,1.14) | 0.693 | 0.98 (0.91,1.07) |
| 14 | IL-5 | 8 | 0.927 | 0.99 (0.88,1.13) | 0.150 | 1.05 (0.98,1.12) | 0.227 | 1.03 (0.98,1.09) | 0.285 | 1.06 (0.96,1.17) | 0.253 | 1.07 (0.97,1.18) |
| 15 | IL-6 | 11 | 0.510 | 1.05 (0.92,1.19) | 0.325 | 1.04 (0.96,1.14) | 0.649 | 1.01 (0.95,1.08) | 0.532 | 0.96 (0.84,1.09) | 0.600 | 1.03 (0.93,1.14) |
| 16 | IL-7 | 13 | 0.857 | 1.01 (0.92,1.10) | 0.067 | 1.04 (1.00,1.09) | 0.455 | 1.02 (0.98,1.06) | 0.197 | 1.05 (0.98,1.12) | 0.083 | 1.05 (0.99,1.10) |
| 17 | IL-8 | 8 | 0.142 | 0.93 (0.86,1.01) | 0.365 | 0.97 (0.91,1.03) | 0.521 | 0.98 (0.93,1.04) | 0.810 | 0.99 (0.90,1.09) | 0.394 | 0.97 (0.91,1.04) |
| 18 | IL-9 | 6 | 0.725 | 1.03 (0.90,1.18) | 0.377 | 1.03 (0.96,1.11) | 0.329 | 1.03 (0.97,1.09) | 0.421 | 1.04 (0.95,1.15) | 0.484 | 1.03 (0.95,1.13) |
| 19 | IL-10 | 15 | 0.104 | 1.10 (0.99,1.23) | 0.088 | 1.05 (0.99,1.12) | 0.294 | 1.03 (0.98,1.08) | 0.501 | 1.04 (0.93,1.16) | 0.127 | 1.06 (1.00,1.12) |
| 20 | IL-12-P70 | 14 | 0.539 | 1.02 (0.95,1.10) | 0.102 | 1.04 (0.99,1.09) | 0.163 | 1.03 (0.99,1.07) | 0.902 | 0.99 (0.90,1.10) | 0.171 | 1.04 (0.99,1.09) |
| 21 | IL-13 | 13 | 0.430 | 1.02 (0.97,1.09) | 0.438 | 1.02 (0.98,1.06) | 0.874 | 1.00 (0.97,1.04) | 0.455 | 0.97 (0.89,1.05) | 0.249 | 1.03 (0.99,1.07) |
| 22 | IL-16 | 9 | 0.546 | 0.98 (0.92,1.04) | 0.918 | 1.00 (0.96,1.04) | 0.728 | 1.01 (0.97,1.05) | 0.653 | 0.98 (0.91,1.06) | 0.705 | 0.99 (0.95,1.03) |
| 23 | IL-17 | 8 | 0.327 | 1.07 (0.94,1.22) | 0.532 | 0.97 (0.89,1.06) | 0.203 | 0.96 (0.90,1.02) | 0.117 | 0.88 (0.75,1.02) | 0.818 | 1.01 (0.89,1.15) |
| 24 | IL-18 | 13 | 0.671 | 0.98 (0.91,1.06) | 0.459 | 1.02 (0.97,1.06) | 0.473 | 1.01 (0.98,1.05) | 0.951 | 1.00 (0.94,1.07) | 0.686 | 1.01 (0.96,1.06) |
| 25 | IP-10 | 11 | 0.380 | 1.04 (0.95,1.15) | 0.967 | 1.00 (0.94,1.07) | 0.856 | 1.00 (0.95,1.04) | 0.912 | 0.99 (0.90,1.09) | 0.964 | 1.00 (0.92,1.09) |
| 26 | M-CSF | 11 | 0.827 | 0.99 (0.93,1.06) | 0.358 | 1.02 (0.98,1.07) | 0.375 | 1.01 (0.98,1.05) | 0.543 | 1.02 (0.96,1.09) | 0.492 | 1.02 (0.96,1.08) |
| 27 | MCP-1-MCAF | 14 | 0.748 | 1.02 (0.89,1.18) | 0.746 | 0.99 (0.93,1.06) | 0.399 | 0.97 (0.91,1.04) | 0.885 | 0.99 (0.89,1.10) | 0.868 | 0.99 (0.93,1.06) |
| 28 | MCP-3 | 6 | 0.655 | 1.02 (0.93,1.12) | 0.882 | 1.00 (0.95,1.05) | 0.786 | 1.01 (0.97,1.04) | 0.759 | 0.99 (0.92,1.06) | 0.756 | 0.99 (0.93,1.05) |
| 29 | MIF | 10 | 0.476 | 1.04 (0.94,1.15) | 0.816 | 1.01 (0.94,1.07) | 0.580 | 1.01 (0.97,1.06) | 0.929 | 1.00 (0.91,1.11) | 0.965 | 1.00 (0.92,1.09) |
| 30 | MIG | 13 | 0.096 | 0.93 (0.87,1.01) | 0.158 | 0.96 (0.92,1.01) | 0.403 | 0.98 (0.95,1.02) | 0.504 | 0.97 (0.89,1.05) | 0.330 | 0.96 (0.89,1.04) |
| 31 | MIP-1A | 6 | 0.989 | 1.00 (0.82,1.22) | 0.394 | 1.04 (0.96,1.12) | 0.290 | 1.04 (0.97,1.11) | 0.307 | 1.08 (0.96,1.21) | 0.254 | 1.08 (0.96,1.21) |
| 32 | MIP-1B | 20 | 0.365 | 1.02 (0.98,1.07) | 0.060 | 1.04 (1.00,1.08) | 0.108 | 1.02 (1.00,1.05) | 0.548 | 1.02 (0.95,1.09) | 0.230 | 1.02 (0.99,1.06) |
| 33 | PDGF-BB | 14 | 0.617 | 0.98 (0.90,1.06) | 0.208 | 0.97 (0.91,1.02) | 0.151 | 0.97 (0.93,1.01) | 0.466 | 0.97 (0.88,1.06) | 0.230 | 0.96 (0.91,1.02) |
| 34 | RANTES | 10 | 0.376 | 1.06 (0.94,1.20) | 0.520 | 1.02 (0.95,1.09) | 0.141 | 1.04 (0.99,1.09) | 0.753 | 1.02 (0.92,1.13) | 0.762 | 1.02 (0.91,1.14) |
| 35 | SCF | 11 | 0.926 | 1.01 (0.86,1.18) | 0.953 | 1.00 (0.93,1.08) | 0.718 | 0.99 (0.93,1.06) | 0.879 | 1.01 (0.90,1.13) | 0.758 | 1.02 (0.92,1.13) |
| 36 | SCGF-B | 18 | 0.832 | 0.99 (0.93,1.06) | 0.738 | 0.99 (0.95,1.04) | 0.753 | 1.01 (0.97,1.04) | 0.814 | 0.99 (0.93,1.06) | 0.859 | 0.99 (0.94,1.05) |
| 37 | SDF-1A | 9 | 0.923 | 0.99 (0.90,1.10) | 0.673 | 0.98 (0.91,1.06) | 0.651 | 0.99 (0.93,1.05) | 0.813 | 0.99 (0.88,1.11) | 0.650 | 0.97 (0.87,1.09) |
| 38 | TNF-A | 4 | 0.550 | 0.97 (0.88,1.06) | 0.209 | 0.96 (0.89,1.02) | 0.185 | 0.96 (0.91,1.02) | 0.346 | 0.95 (0.87,1.04) | 0.366 | 0.95 (0.88,1.03) |
| 39 | TNF-B | 5 | 0.744 | 1.01 (0.95,1.08) | 0.809 | 1.00 (0.97,1.04) | 0.580 | 0.99 (0.95,1.03) | 0.870 | 1.01 (0.93,1.09) | 0.660 | 1.01 (0.97,1.05) |
| 40 | TRAIL | 16 | 0.144 | 1.03 (0.99,1.07) | 0.466 | 1.02 (0.98,1.06) | 0.297 | 1.02 (0.99,1.05) | 0.275 | 0.96 (0.90,1.03) | 0.263 | 1.02 (0.98,1.07) |
| 41 | VEGF | 15 | 0.325 | 1.03 (0.97,1.09) | 0.094 | 1.03 (0.99,1.07) | 0.123 | 1.03 (0.99,1.07) | 0.835 | 1.01 (0.94,1.08) | 0.166 | 1.03 (0.99,1.06) |

**Table S2. Heterogeneity and horizontal pleiotropy tests of forty-one inflammatory cytokines on KOA.**

| **N** | **Exposures** | **Q_1_ pval** | **Q_2_ pval** | **intercept** | **intercept pval** | **MR-PRESSO**  **pval** | **Recommended Method** |
| --- | --- | --- | --- | --- | --- | --- | --- |
| 1 | B-NGF | 0.286 | 0.634 | -0.054 | 0.232 | 0.39 | IVW |
| 2 | CTACK | 0.477 | 0.441 | 0.006 | 0.458 | 0.41 | IVW |
| 3 | EOTAXIN | 0.129 | 0.096 | -0.001 | 0.903 | 0.14 | IVW |
| 4 | FGF-BASIC | 0.612 | 0.505 | -0.005 | 0.703 | 0.75 | IVW |
| 5 | G-CSF | 0.597 | 0.763 | -0.010 | 0.173 | 0.68 | IVW |
| 6 | GROA | 0.467 | 0.706 | -0.021 | 0.100 | 0.55 | IVW |
| 7 | HGF | 0.602 | 0.736 | 0.014 | 0.197 | 0.67 | IVW |
| 8 | IFN-G | 0.346 | 0.311 | 0.006 | 0.483 | 0.43 | IVW |
| 9 | IL-1B | 0.171 | 0.142 | 0.034 | 0.571 | / | IVW |
| 10 | IL-1RA | 0.032 | 0.040 | 0.016 | 0.334 | 0.02 | My-Egger |
| 11 | IL-2 | 0.064 | 0.105 | 0.014 | 0.249 | 0.14 | IVW |
| 12 | IL-2RA | 0.175 | 0.320 | -0.014 | 0.147 | 0.33 | IVW |
| 13 | IL-4 | 0.291 | 0.278 | 0.006 | 0.404 | 0.47 | IVW |
| 14 | IL-5 | 0.829 | 0.792 | 0.008 | 0.541 | 0.88 | IVW |
| 15 | IL-6 | 0.404 | 0.342 | -0.004 | 0.604 | 0.36 | IVW |
| 16 | IL-7 | 0.090 | 0.063 | 0.002 | 0.859 | 0.12 | IVW |
| 17 | IL-8 | 0.071 | 0.167 | 0.016 | 0.159 | 0.15 | IVW |
| 18 | IL-9 | 0.828 | 0.708 | 0.000 | 0.984 | 0.86 | IVW |
| 19 | IL-10 | 0.182 | 0.243 | -0.010 | 0.183 | 0.23 | IVW |
| 20 | IL-12-P70 | 0.308 | 0.244 | 0.001 | 0.874 | 0.37 | IVW |
| 21 | IL-13 | 0.264 | 0.254 | -0.007 | 0.394 | 0.25 | IVW |
| 22 | IL-16 | 0.119 | 0.144 | 0.011 | 0.303 | 0.19 | IVW |
| 23 | IL-17 | 0.489 | 0.878 | -0.021 | 0.091 | 0.44 | IVW |
| 24 | IL-18 | 0.096 | 0.097 | 0.009 | 0.378 | 0.15 | IVW |
| 25 | IP-10 | 0.751 | 0.800 | -0.010 | 0.276 | 0.78 | IVW |
| 26 | M-CSF | 0.632 | 0.592 | 0.008 | 0.483 | 0.64 | IVW |
| 27 | MCP-1-MCAF | 0.054 | 0.051 | -0.009 | 0.443 | 0.08 | IVW |
| 28 | MCP-3 | 0.586 | 0.465 | -0.005 | 0.705 | 0.61 | IVW |
| 29 | MIF | 0.685 | 0.621 | -0.005 | 0.596 | 0.68 | IVW |
| 30 | MIG | 0.744 | 0.880 | 0.016 | 0.134 | 0.79 | IVW |
| 31 | MIP-1A | 0.894 | 0.822 | 0.006 | 0.737 | 0.93 | IVW |
| 32 | MIP-1B | 0.466 | 0.402 | 0.001 | 0.927 | 0.56 | IVW |
| 33 | PDGF-BB | 0.727 | 0.658 | -0.002 | 0.793 | 0.82 | IVW |
| 34 | RANTES | 0.639 | 0.554 | -0.005 | 0.712 | 0.64 | IVW |
| 35 | SCF | 0.251 | 0.190 | -0.003 | 0.787 | 0.30 | IVW |
| 36 | SCGF-B | 0.224 | 0.187 | 0.004 | 0.657 | 0.31 | IVW |
| 37 | SDF-1A | 0.992 | 0.983 | -0.002 | 0.829 | 0.99 | IVW |
| 38 | TNF-A | 0.991 | 0.968 | -0.003 | 0.860 | 0.99 | IVW |
| 39 | TNF-B | 0.137 | 0.152 | -0.011 | 0.398 | 0.25 | IVW |
| 40 | TRAIL | 0.225 | 0.250 | -0.007 | 0.266 | 0.20 | IVW |
| 41 | VEGF | 0.076 | 0.054 | -0.001 | 0.913 | 0.16 | IVW |

Q1 pval: p value of Q test from IVW method; Q2 pval: p value of Q test from MR-Egger method
Abbreviations: pval, p-value; Q, Cochran Q statistics; SNPs, single nucleotide polymorphisms; IVW, the inverse variance weighted method;

**Table S3. SNPs information of forty-one inflammation cytokines with KOA**

| **SNP** | **inflammatory cytokines** | | | | | **KOA** | | | | |
| --- | --- | --- | --- | --- | --- | --- | --- | --- | --- | --- |
|  | **effect allele** | **other allele** | **Beta** | **se** | ***p*val** | ***F*** | **Beta** | **se** | ***p*val** | **R^2^** |
| **bNGF** |  |  |  |  |  |  |  |  |  |  |
| rs28637706 | T | G | -0.1554 | 0.0261 | 2.717E-09 | 35.431 | -0.0047 | 0.0095 | 0.625 | 0.010 |
| rs71641308 | T | C | 0.1969 | 0.0429 | 0.000004424 | 21.054 | 0.0244 | 0.0175 | 0.163 | 0.006 |
| rs73472576 | T | C | -0.1146 | 0.0251 | 0.000004813 | 20.834 | 0.0116 | 0.0096 | 0.227 | 0.006 |
| rs7970581 | T | G | 0.1358 | 0.028 | 0.000001223 | 23.509 | 0.0091 | 0.0109 | 0.400 | 0.007 |
| **CTACK** |  |  |  |  |  |  |  |  |  |  |
| rs116303454 | A | G | 0.3754 | 0.081 | 0.000003579 | 21.468 | -0.0587 | 0.032 | 0.067 | 0.006 |
| rs118084576 | A | G | 0.5675 | 0.1226 | 0.00000366 | 21.415 | -0.033 | 0.0425 | 0.438 | 0.006 |
| rs135564 | A | G | -0.1672 | 0.0267 | 3.593E-10 | 39.193 | -0.0043 | 0.0102 | 0.672 | 0.011 |
| rs141331414 | A | G | 0.1977 | 0.0415 | 0.000001886 | 22.682 | 0.0061 | 0.0177 | 0.730 | 0.006 |
| rs2070074 | A | G | 0.4401 | 0.0372 | 2.596E-32 | 139.888 | 0.0187 | 0.0158 | 0.238 | 0.037 |
| rs55764737 | T | C | 0.5424 | 0.0967 | 2.012E-08 | 31.445 | -0.0412 | 0.025 | 0.100 | 0.008 |
| rs57338032 | A | G | 0.1443 | 0.0316 | 0.000004831 | 20.841 | -0.0082 | 0.0125 | 0.512 | 0.006 |
| rs57789542 | T | C | -0.7687 | 0.1659 | 0.000003575 | 21.458 | -0.0148 | 0.0324 | 0.649 | 0.006 |
| rs60247384 | T | C | 0.1128 | 0.0245 | 0.000004302 | 21.186 | 0.0129 | 0.0107 | 0.228 | 0.006 |
| rs62578137 | T | C | -0.1311 | 0.0286 | 0.000004658 | 21.001 | 0.0001 | 0.0121 | 0.994 | 0.006 |
| rs72729450 | T | C | -0.5123 | 0.1094 | 0.000002814 | 21.917 | 0.0267 | 0.0376 | 0.478 | 0.006 |
| rs7333764 | T | C | 0.2811 | 0.0591 | 0.000002002 | 22.610 | 0.0096 | 0.0335 | 0.775 | 0.006 |
| rs76395525 | A | G | 0.5193 | 0.1081 | 0.000001553 | 23.065 | 0.031 | 0.041 | 0.450 | 0.006 |
| **EOTAXIN** |  |  |  |  |  |  |  |  |  |  |
| rs11087905 | A | C | 0.0954 | 0.0188 | 0.000000407 | 25.744 | 0.012 | 0.0109 | 0.271 | 0.003 |
| rs112347425 | T | C | 0.1595 | 0.0276 | 7.771E-09 | 33.389 | -0.0084 | 0.0157 | 0.592 | 0.004 |
| rs12075 | A | G | 0.1692 | 0.0155 | 1.211E-27 | 119.133 | -0.0028 | 0.0094 | 0.768 | 0.014 |
| rs147287945 | A | G | -0.1512 | 0.0313 | 0.000001356 | 23.330 | -0.0356 | 0.0173 | 0.039 | 0.003 |
| rs187131 | C | G | 0.1264 | 0.0253 | 5.742E-07 | 24.954 | 0.0243 | 0.0141 | 0.086 | 0.003 |
| rs2024050 | A | G | 0.164 | 0.0302 | 5.467E-08 | 29.483 | -0.0143 | 0.0152 | 0.349 | 0.004 |
| rs2027855 | T | C | 0.0743 | 0.0162 | 0.000004272 | 21.030 | -0.0007 | 0.0098 | 0.947 | 0.003 |
| rs2211994 | T | C | 0.0876 | 0.0177 | 6.981E-07 | 24.488 | 0.0038 | 0.0105 | 0.719 | 0.003 |
| rs2228467 | T | C | -0.4154 | 0.0291 | 3.473E-46 | 203.723 | -0.0085 | 0.0193 | 0.661 | 0.024 |
| rs5754733 | A | C | -0.105 | 0.0213 | 8.196E-07 | 24.295 | 0.0012 | 0.011 | 0.911 | 0.003 |
| rs59808887 | T | C | -0.1698 | 0.0356 | 0.000001886 | 22.744 | -0.0071 | 0.0175 | 0.685 | 0.003 |
| rs745331 | A | G | -0.0821 | 0.0176 | 0.000003036 | 21.755 | 0.0235 | 0.0102 | 0.021 | 0.003 |
| rs75426604 | A | C | -0.1371 | 0.0291 | 0.000002397 | 22.191 | -0.0082 | 0.0137 | 0.548 | 0.003 |
| rs79722574 | T | C | -0.1092 | 0.0227 | 0.000001497 | 23.136 | 0.0122 | 0.0127 | 0.336 | 0.003 |
| rs80341932 | A | G | 0.101 | 0.0204 | 7.403E-07 | 24.506 | 0.0108 | 0.0107 | 0.316 | 0.003 |
| rs9317045 | A | C | 0.1172 | 0.0236 | 6.954E-07 | 24.656 | 0.027 | 0.0127 | 0.033 | 0.003 |
| **FGF-BASIC** |  |  |  |  |  |  |  |  |  |  |
| rs116745220 | A | G | -0.6176 | 0.1324 | 0.000003085 | 21.753 | 0.0369 | 0.9709 | 21.753 | 0.003 |
| rs13412535 | A | G | -0.1129 | 0.0224 | 4.763E-07 | 25.397 | 0.011 | 0.3141 | 25.397 | 0.003 |
| rs145577605 | A | G | 0.2043 | 0.0427 | 0.000001672 | 22.886 | 0.0592 | 0.4876 | 22.886 | 0.003 |
| rs61990749 | C | G | 0.1124 | 0.0228 | 8.225E-07 | 24.297 | 0.0147 | 0.1724 | 24.297 | 0.003 |
| rs75168112 | T | C | -0.1024 | 0.0214 | 0.000001637 | 22.891 | 0.014 | 0.2902 | 22.891 | 0.003 |
| rs78873483 | A | G | 0.1286 | 0.0282 | 0.00000498 | 20.791 | 0.015 | 0.786799 | 20.791 | 0.003 |
| rs9903590 | T | C | 0.1281 | 0.0267 | 0.000001622 | 23.012 | 0.0158 | 0.8989 | 23.012 | 0.003 |
| **G-CSF** |  |  |  |  |  |  |  |  |  |  |
| rs115256310 | A | G | -0.6788 | 0.1359 | 0.000000585 | 24.942 | 0.0007 | 0.0369 | 0.985 | 0.003 |
| rs117261691 | T | C | 0.1318 | 0.0288 | 0.000004669 | 20.938 | -0.0132 | 0.034 | 0.698 | 0.003 |
| rs11903143 | A | G | 0.0889 | 0.0175 | 3.784E-07 | 25.800 | -0.0153 | 0.0104 | 0.142 | 0.003 |
| rs145756094 | C | G | -0.7323 | 0.1479 | 7.399E-07 | 24.508 | 0.0357 | 0.0367 | 0.331 | 0.004 |
| rs2671444 | A | G | -0.0776 | 0.0166 | 0.000002861 | 21.847 | 0.0017 | 0.0096 | 0.859 | 0.003 |
| rs586313 | T | C | -0.0883 | 0.0187 | 0.000002355 | 22.291 | 0.0123 | 0.0107 | 0.250 | 0.003 |
| rs74148555 | T | C | -0.3771 | 0.0753 | 5.591E-07 | 25.073 | 0.0196 | 0.0272 | 0.470 | 0.003 |
| rs76287671 | T | C | 0.0894 | 0.0189 | 0.000002191 | 22.369 | -0.0191 | 0.0118 | 0.108 | 0.003 |
| rs77318030 | T | C | -0.2031 | 0.0427 | 0.000002017 | 22.618 | 0.0447 | 0.0207 | 0.031 | 0.003 |
| **GROA** |  |  |  |  |  |  |  |  |  |  |
| rs1113500 | T | G | 0.1162 | 0.0243 | 0.000001721 | 22.854 | -0.0148 | 0.0097 | 0.126 | 0.006 |
| rs114991247 | T | C | -0.2202 | 0.0463 | 0.000001971 | 22.606 | 0.0183 | 0.0328 | 0.577 | 0.006 |
| rs118158560 | A | G | 0.2761 | 0.0592 | 0.000003085 | 21.739 | -0.0304 | 0.0198 | 0.125 | 0.006 |
| rs12075 | A | G | 0.3724 | 0.0236 | 3.459E-56 | 248.857 | -0.0028 | 0.0094 | 0.768 | 0.066 |
| rs140734053 | A | G | 0.7333 | 0.1545 | 0.000002069 | 22.514 | 0.0262 | 0.036 | 0.467 | 0.006 |
| rs185768063 | A | G | 0.4038 | 0.076 | 1.055E-07 | 28.214 | -0.0051 | 0.0499 | 0.918 | 0.008 |
| rs188345231 | T | C | 0.6177 | 0.1322 | 0.000002968 | 21.820 | -0.0427 | 0.0341 | 0.210 | 0.006 |
| rs508977 | T | G | -0.3838 | 0.0279 | 4.566E-43 | 189.128 | -0.0111 | 0.0109 | 0.306 | 0.051 |
| rs62024303 | A | G | -0.3013 | 0.066 | 0.000004908 | 20.829 | 0.0029 | 0.0227 | 0.897 | 0.006 |
| rs76390238 | C | G | 0.6223 | 0.1352 | 0.000004141 | 21.174 | -0.0098 | 0.0327 | 0.765 | 0.006 |
| rs78653452 | T | G | -0.7395 | 0.1559 | 0.000002093 | 22.487 | -0.0488 | 0.0411 | 0.235 | 0.006 |
| **HGF** |  |  |  |  |  |  |  |  |  |  |
| rs11060254 | A | G | -0.0765 | 0.0166 | 0.000003974 | 21.233 | 0.0033 | 0.0097 | 0.735 | 0.003 |
| rs13412535 | A | G | -0.1043 | 0.0213 | 9.671E-07 | 23.972 | -0.0111 | 0.011 | 0.314 | 0.003 |
| rs1617833 | C | G | -0.0749 | 0.016 | 0.000002925 | 21.909 | -0.0164 | 0.0095 | 0.082 | 0.003 |
| rs180840563 | A | T | -0.2022 | 0.0416 | 0.000001153 | 23.620 | -0.0033 | 0.0318 | 0.917 | 0.003 |
| rs2003620 | T | C | 0.2277 | 0.0487 | 0.000002978 | 21.856 | -0.009 | 0.0203 | 0.658 | 0.003 |
| rs3748034 | T | G | 0.1529 | 0.0233 | 5.21E-11 | 43.053 | -0.0171 | 0.0133 | 0.198 | 0.005 |
| rs4245058 | T | C | -0.1552 | 0.0331 | 0.000002683 | 21.980 | 0.0047 | 0.0158 | 0.764 | 0.003 |
| rs57146176 | A | G | -0.0987 | 0.0208 | 0.000002184 | 22.511 | 0.0069 | 0.0202 | 0.734 | 0.003 |
| rs5745687 | T | C | -0.3008 | 0.0404 | 9.922E-14 | 55.423 | 0.0125 | 0.0188 | 0.506 | 0.007 |
| **IFN-G** |  |  |  |  |  |  |  |  |  |  |
| rs10481651 | A | G | -0.0793 | 0.0168 | 0.000002183 | 22.275 | -0.0083 | 0.0099 | 0.401 | 0.003 |
| rs10761731 | A | T | -0.0813 | 0.0167 | 0.000001068 | 23.694 | 0.0018 | 0.0094 | 0.850 | 0.003 |
| rs113600793 | A | C | 0.1871 | 0.0371 | 4.426E-07 | 25.427 | 0.0031 | 0.024 | 0.896 | 0.003 |
| rs115729819 | A | G | 0.2511 | 0.0514 | 0.000001045 | 23.859 | -0.0931 | 0.0389 | 0.017 | 0.003 |
| rs11843756 | T | G | 0.1812 | 0.0391 | 0.000003622 | 21.471 | -0.0466 | 0.0293 | 0.112 | 0.003 |
| rs12420286 | T | C | 0.2357 | 0.05 | 0.000002452 | 22.216 | -0.0066 | 0.0229 | 0.774 | 0.003 |
| rs2073438 | A | G | 0.092 | 0.0188 | 9.551E-07 | 23.941 | -0.0012 | 0.0102 | 0.904 | 0.003 |
| rs2188420 | C | G | 0.1005 | 0.0201 | 5.898E-07 | 24.994 | -0.0099 | 0.0143 | 0.489 | 0.003 |
| rs60059008 | A | G | 0.0852 | 0.0176 | 0.000001301 | 23.428 | -0.0006 | 0.0098 | 0.953 | 0.003 |
| rs73479333 | C | G | -0.1123 | 0.024 | 0.000002816 | 21.889 | -0.0276 | 0.0178 | 0.121 | 0.003 |
| rs74148555 | T | C | -0.3771 | 0.077 | 9.858E-07 | 23.978 | 0.0196 | 0.0272 | 0.470 | 0.003 |
| rs78296352 | T | G | 0.3419 | 0.065 | 0.000000142 | 27.660 | 0.0109 | 0.024 | 0.649 | 0.004 |
| **IL-1B** |  |  |  |  |  |  |  |  |  |  |
| rs143319329 | T | C | 0.4357 | 0.093 | 0.000002835 | 21.936 | -0.0071 | 0.0296 | 0.810 | 0.007 |
| rs61335305 | A | C | 0.4333 | 0.0928 | 0.000003015 | 21.788 | -0.074 | 0.0349 | 0.034 | 0.006 |
| rs62015704 | A | G | 0.1786 | 0.0372 | 0.000001624 | 23.037 | 0.0055 | 0.0141 | 0.695 | 0.007 |
| **IL-1RA** |  |  |  |  |  |  |  |  |  |  |
| rs1054402 | T | C | 0.1325 | 0.0269 | 8.201E-07 | 24.249 | -0.0176 | 0.0108 | 0.103 | 0.007 |
| rs11627423 | A | C | 0.1178 | 0.0246 | 0.00000165 | 22.918 | -0.0108 | 0.0095 | 0.253 | 0.006 |
| rs11869294 | C | G | -0.2286 | 0.047 | 0.000001128 | 23.644 | -0.036 | 0.0272 | 0.186 | 0.006 |
| rs147747784 | C | G | 0.3582 | 0.0754 | 0.000002039 | 22.557 | -0.0576 | 0.0269 | 0.032 | 0.006 |
| rs187166731 | T | C | -0.2424 | 0.0504 | 0.000001547 | 23.119 | 0.0267 | 0.0676 | 0.693 | 0.006 |
| rs3876037 | A | G | 0.1234 | 0.027 | 0.000004733 | 20.877 | -0.0099 | 0.01 | 0.326 | 0.006 |
| rs4441609 | T | C | 0.1056 | 0.0231 | 0.000004746 | 20.887 | 0.0141 | 0.0096 | 0.139 | 0.006 |
| rs56134659 | A | G | -0.1109 | 0.0236 | 0.000002564 | 22.070 | 0.0002 | 0.0102 | 0.983 | 0.006 |
| rs61335305 | A | C | 0.4333 | 0.0928 | 0.000003015 | 21.788 | -0.074 | 0.0349 | 0.034 | 0.006 |
| rs6699436 | A | G | -0.1858 | 0.0404 | 0.000004365 | 21.139 | -0.0208 | 0.0133 | 0.118 | 0.006 |
| **IL-2** |  |  |  |  |  |  |  |  |  |  |
| rs13412535 | A | G | -0.1043 | 0.0213 | 9.671E-07 | 23.972 | -0.0111 | 0.011 | 0.314 | 0.003 |
| rs16836080 | A | G | 0.1158 | 0.0253 | 0.000004841 | 20.938 | 0.0127 | 0.0103 | 0.218 | 0.006 |
| rs170117 | T | C | -0.1637 | 0.0347 | 0.000002442 | 22.243 | -0.0058 | 0.0138 | 0.676 | 0.006 |
| rs2690020 | A | G | 0.1158 | 0.0245 | 0.000002273 | 22.327 | -0.0012 | 0.0095 | 0.902 | 0.006 |
| rs4634519 | A | G | -0.1249 | 0.0268 | 0.00000318 | 21.707 | 0.0161 | 0.0103 | 0.116 | 0.006 |
| rs61335305 | A | C | 0.4333 | 0.0928 | 0.000003015 | 21.788 | -0.074 | 0.0349 | 0.034 | 0.006 |
| rs62124990 | T | G | -0.7013 | 0.149 | 0.000002502 | 22.141 | 0.0246 | 0.0297 | 0.408 | 0.006 |
| rs7615304 | A | G | -0.1139 | 0.024 | 0.000002161 | 22.510 | -0.0194 | 0.0097 | 0.045 | 0.006 |
| **IL-2RA** |  |  |  |  |  |  |  |  |  |  |
| rs11241559 | T | G | -0.124 | 0.0264 | 0.00000275 | 22.050 | 0.0169 | 0.011 | 0.123 | 0.006 |
| rs115360066 | A | G | 0.1776 | 0.0377 | 0.000002418 | 22.180 | -0.0203 | 0.0157 | 0.198 | 0.006 |
| rs117244812 | A | G | -0.7187 | 0.1493 | 0.000001474 | 23.160 | -0.0537 | 0.0438 | 0.220 | 0.006 |
| rs12722497 | A | C | 0.6287 | 0.0482 | 7.978E-39 | 170.043 | -0.0073 | 0.0165 | 0.659 | 0.044 |
| rs12799226 | T | C | -0.1285 | 0.0277 | 0.000003561 | 21.509 | 0.0023 | 0.0114 | 0.841 | 0.006 |
| rs185231391 | T | C | 0.8568 | 0.1803 | 0.000002004 | 22.570 | -0.0571 | 0.0425 | 0.180 | 0.006 |
| rs28441585 | A | T | 0.1269 | 0.0271 | 0.000002934 | 21.915 | -0.0254 | 0.0112 | 0.023 | 0.006 |
| rs4733117 | A | C | 0.1439 | 0.0291 | 7.912E-07 | 24.440 | 0.0014 | 0.0133 | 0.915 | 0.007 |
| **IL-4** |  |  |  |  |  |  |  |  |  |  |
| rs10512267 | T | C | -0.0824 | 0.016 | 2.734E-07 | 26.516 | -0.0065 | 0.0099 | 0.514 | 0.003 |
| rs116705532 | T | G | -0.4675 | 0.0978 | 0.000001727 | 22.844 | 0.0526 | 0.0366 | 0.150 | 0.003 |
| rs117146485 | T | C | -0.2856 | 0.0625 | 0.000004945 | 20.876 | -0.0219 | 0.0468 | 0.640 | 0.003 |
| rs12238729 | T | C | 0.5271 | 0.1096 | 0.000001505 | 23.116 | 0.0023 | 0.0414 | 0.956 | 0.006 |
| rs13106889 | A | T | -0.1186 | 0.0224 | 1.215E-07 | 28.026 | -0.0011 | 0.0128 | 0.935 | 0.003 |
| rs17713451 | A | G | 0.1255 | 0.0252 | 0.000000641 | 24.796 | 0.001 | 0.0135 | 0.942 | 0.003 |
| rs2073438 | A | G | 0.092 | 0.0188 | 9.551E-07 | 23.941 | -0.0012 | 0.0102 | 0.904 | 0.003 |
| rs6765768 | A | G | 0.0796 | 0.0167 | 0.000001853 | 22.714 | 0.0194 | 0.0096 | 0.043 | 0.003 |
| rs6969391 | T | C | 0.0767 | 0.0166 | 0.000003587 | 21.344 | -0.0281 | 0.0116 | 0.015 | 0.003 |
| rs73023729 | A | G | -0.1796 | 0.0365 | 8.561E-07 | 24.206 | -0.0147 | 0.037 | 0.691 | 0.003 |
| rs7613691 | A | G | 0.1787 | 0.0382 | 0.000002962 | 21.878 | -0.0259 | 0.019 | 0.174 | 0.003 |
| rs79597994 | T | C | -0.5855 | 0.1271 | 0.000004056 | 21.216 | 0.0204 | 0.0298 | 0.493 | 0.003 |
| rs9508291 | T | C | -0.168 | 0.0358 | 0.000002669 | 22.016 | 0.0135 | 0.0187 | 0.471 | 0.003 |
| rs9941733 | A | G | 0.1156 | 0.0229 | 4.331E-07 | 25.476 | 0.0072 | 0.0124 | 0.562 | 0.003 |
| **IL-5** |  |  |  |  |  |  |  |  |  |  |
| rs11680908 | A | G | 0.2593 | 0.0552 | 0.000002617 | 22.053 | 0.0107 | 0.019 | 0.573 | 0.006 |
| rs148634917 | A | G | -0.517 | 0.1087 | 0.000001974 | 22.608 | -0.0387 | 0.0346 | 0.264 | 0.007 |
| rs28793375 | T | C | 0.1697 | 0.0362 | 0.000002746 | 21.963 | 0.0096 | 0.0139 | 0.490 | 0.006 |
| rs6737109 | T | C | 0.1135 | 0.0246 | 0.000003806 | 21.275 | 0.0055 | 0.0094 | 0.562 | 0.006 |
| rs72831687 | A | G | -0.5337 | 0.1104 | 0.000001324 | 23.356 | 0.0319 | 0.0523 | 0.541 | 0.007 |
| rs73040130 | T | C | 0.2745 | 0.0525 | 1.709E-07 | 27.322 | -0.0094 | 0.0196 | 0.631 | 0.008 |
| rs74811276 | A | G | 0.217 | 0.0471 | 0.000004082 | 21.214 | -0.0058 | 0.0173 | 0.737 | 0.006 |
| rs9472168 | A | G | 0.1568 | 0.0253 | 5.423E-10 | 38.388 | 0.013 | 0.0094 | 0.167 | 0.011 |
| **IL-6** |  |  |  |  |  |  |  |  |  |  |
| rs10752777 | A | T | 0.1083 | 0.0235 | 0.000004171 | 21.233 | -0.0084 | 0.0172 | 0.626 | 0.003 |
| rs10982213 | A | G | -0.0849 | 0.0176 | 0.000001353 | 23.264 | 0.0022 | 0.0111 | 0.843 | 0.003 |
| rs113098456 | A | G | -0.1553 | 0.0339 | 0.000004641 | 20.982 | -0.0284 | 0.0189 | 0.134 | 0.003 |
| rs1333040 | T | C | 0.0747 | 0.0157 | 0.000001993 | 22.633 | -0.0057 | 0.0094 | 0.547 | 0.003 |
| rs13412535 | A | G | -0.1043 | 0.0213 | 9.671E-07 | 23.972 | -0.0111 | 0.011 | 0.314 | 0.003 |
| rs2404476 | A | G | 0.0734 | 0.0156 | 0.000002684 | 22.133 | 0.0138 | 0.0093 | 0.138 | 0.003 |
| rs4684700 | T | C | -0.0747 | 0.0162 | 0.000003912 | 21.257 | 0.0167 | 0.0094 | 0.076 | 0.003 |
| rs72831623 | A | G | 0.197 | 0.0369 | 9.29E-08 | 28.495 | -0.0079 | 0.0216 | 0.714 | 0.003 |
| rs73273528 | T | C | 0.268 | 0.0553 | 0.00000125 | 23.481 | -0.0199 | 0.0258 | 0.439 | 0.003 |
| rs75101555 | C | G | -0.3625 | 0.0781 | 0.000003439 | 21.538 | -0.0156 | 0.0294 | 0.596 | 0.003 |
| rs76856708 | T | C | 0.336 | 0.0697 | 0.000001427 | 23.233 | 0.0147 | 0.0247 | 0.552 | 0.003 |
| **IL-7** |  |  |  |  |  |  |  |  |  |  |
| rs10196226 | A | G | 0.1538 | 0.0327 | 0.000002501 | 22.109 | 0.0197 | 0.0145 | 0.174 | 0.006 |
| rs115215018 | T | C | 0.5985 | 0.1308 | 0.000004755 | 20.925 | 0.0143 | 0.0351 | 0.684 | 0.006 |
| rs117509142 | T | C | -0.3213 | 0.0684 | 0.000002599 | 22.052 | 0.0455 | 0.0246 | 0.064 | 0.006 |
| rs141425475 | T | C | -0.4801 | 0.1018 | 0.00000239 | 22.229 | -0.0386 | 0.0292 | 0.187 | 0.006 |
| rs147747784 | C | G | 0.3582 | 0.0754 | 0.000002039 | 22.557 | -0.0576 | 0.0269 | 0.032 | 0.006 |
| rs17091524 | T | C | 0.5092 | 0.1015 | 5.244E-07 | 25.153 | 0.0158 | 0.0274 | 0.565 | 0.007 |
| rs1958987 | T | C | 0.1261 | 0.0263 | 0.000001604 | 22.976 | 0.0029 | 0.0099 | 0.774 | 0.007 |
| rs218247 | C | G | -0.1343 | 0.0285 | 0.000002419 | 22.193 | -0.007 | 0.0118 | 0.553 | 0.006 |
| rs28793375 | T | C | 0.1697 | 0.0362 | 0.000002746 | 21.963 | 0.0096 | 0.0139 | 0.490 | 0.006 |
| rs62006410 | T | C | -0.1492 | 0.0302 | 7.588E-07 | 24.393 | 0.0135 | 0.0116 | 0.243 | 0.007 |
| rs6921438 | A | G | -0.3204 | 0.0246 | 8.707E-39 | 169.536 | -0.0156 | 0.0093 | 0.094 | 0.047 |
| rs77981494 | T | C | -0.5201 | 0.1055 | 8.225E-07 | 24.289 | -0.0313 | 0.0329 | 0.340 | 0.007 |
| rs78346957 | A | G | 0.4632 | 0.1008 | 0.0000043 | 21.104 | -0.0476 | 0.0361 | 0.188 | 0.006 |
| **IL-8** |  |  |  |  |  |  |  |  |  |  |
| rs116726256 | T | C | -0.2247 | 0.0489 | 0.000004261 | 21.103 | 0.0693 | 0.0369 | 0.061 | 0.006 |
| rs12075 | A | G | 0.3724 | 0.0236 | 3.459E-56 | 248.857 | -0.0028 | 0.0094 | 0.768 | 0.066 |
| rs12438669 | A | C | -0.1182 | 0.0252 | 0.000002597 | 21.988 | -0.0094 | 0.0097 | 0.331 | 0.006 |
| rs141926526 | A | C | -0.6221 | 0.1308 | 0.00000196 | 22.608 | 0.0329 | 0.0239 | 0.169 | 0.006 |
| rs183628733 | T | C | 0.6547 | 0.1417 | 0.000003821 | 21.335 | -0.0653 | 0.0352 | 0.064 | 0.006 |
| rs2673604 | A | C | -0.118 | 0.0254 | 0.000003289 | 21.570 | -0.0197 | 0.0103 | 0.056 | 0.006 |
| rs3786107 | A | G | 0.2463 | 0.0517 | 0.000001935 | 22.683 | 0.0009 | 0.0206 | 0.966 | 0.006 |
| rs75840288 | A | C | 0.5125 | 0.1121 | 0.000004846 | 20.890 | 0.0126 | 0.0241 | 0.600 | 0.006 |
| **IL-9** |  |  |  |  |  |  |  |  |  |  |
| rs117807175 | C | G | -0.5225 | 0.1106 | 0.000002327 | 22.306 | -0.0246 | 0.0283 | 0.384 | 0.006 |
| rs3736858 | C | G | -0.1351 | 0.0291 | 0.000003373 | 21.542 | -0.0082 | 0.0129 | 0.527 | 0.006 |
| rs41294750 | T | C | 0.3442 | 0.0736 | 0.000002916 | 21.859 | 0.0313 | 0.0279 | 0.261 | 0.006 |
| rs4880409 | T | C | -0.3552 | 0.0716 | 6.952E-07 | 24.597 | 0.0224 | 0.0301 | 0.458 | 0.007 |
| rs73443903 | A | C | 0.2162 | 0.046 | 0.000002569 | 22.078 | 0.0002 | 0.0161 | 0.989 | 0.006 |
| rs76963786 | T | C | -0.2856 | 0.0556 | 2.775E-07 | 26.371 | -0.0058 | 0.016 | 0.715 | 0.007 |
| **IL-10** |  |  |  |  |  |  |  |  |  |  |
| rs10457128 | A | G | -0.0854 | 0.0172 | 6.956E-07 | 24.646 | -0.0124 | 0.0096 | 0.198 | 0.003 |
| rs10493718 | A | C | -0.1081 | 0.0222 | 0.000001068 | 23.705 | -0.0069 | 0.0111 | 0.537 | 0.003 |
| rs10888839 | C | G | 0.1203 | 0.025 | 0.000001562 | 23.149 | -0.0022 | 0.0173 | 0.899 | 0.003 |
| rs1530455 | T | C | 0.082 | 0.0174 | 0.000002527 | 22.203 | -0.0132 | 0.0095 | 0.166 | 0.003 |
| rs2086656 | T | C | -0.08 | 0.017 | 0.000002589 | 22.140 | 0.0095 | 0.0102 | 0.352 | 0.003 |
| rs282258 | T | C | 0.0993 | 0.0162 | 8.63E-10 | 37.563 | 0.0139 | 0.0094 | 0.138 | 0.005 |
| rs3002131 | C | G | 0.1191 | 0.026 | 0.000004592 | 20.978 | 0.0176 | 0.0144 | 0.222 | 0.003 |
| rs3025021 | T | C | 0.0913 | 0.0194 | 0.000002609 | 22.142 | -0.002 | 0.0101 | 0.841 | 0.003 |
| rs339203 | T | C | 0.0954 | 0.0203 | 0.000002754 | 22.078 | 0.004 | 0.0113 | 0.725 | 0.004 |
| rs383684 | A | G | 0.092 | 0.0197 | 0.000003168 | 21.804 | -0.037 | 0.0148 | 0.013 | 0.003 |
| rs41282660 | A | G | -0.1169 | 0.0254 | 0.000004234 | 21.176 | -0.0033 | 0.0145 | 0.820 | 0.003 |
| rs6085948 | A | G | 0.0977 | 0.0202 | 0.00000128 | 23.387 | 0.0062 | 0.011 | 0.571 | 0.003 |
| rs6799107 | T | C | -0.095 | 0.0206 | 0.000003992 | 21.262 | 0.0152 | 0.0114 | 0.182 | 0.003 |
| rs6921438 | A | G | -0.3204 | 0.0246 | 8.707E-39 | 169.536 | -0.0156 | 0.0093 | 0.094 | 0.047 |
| rs7088799 | T | G | -0.0815 | 0.0166 | 9.352E-07 | 24.098 | 0.0029 | 0.0094 | 0.757 | 0.003 |
| **IL-12-P70** |  |  |  |  |  |  |  |  |  |  |
| rs10761731 | A | T | -0.0813 | 0.0167 | 0.000001068 | 23.694 | 0.0018 | 0.0094 | 0.850 | 0.003 |
| rs13209117 | A | G | 0.0981 | 0.0186 | 1.271E-07 | 27.810 | 0.0023 | 0.0105 | 0.824 | 0.003 |
| rs2123852 | T | C | 0.0942 | 0.0204 | 0.00000373 | 21.318 | 0.0077 | 0.013 | 0.556 | 0.003 |
| rs273702 | A | G | -0.127 | 0.027 | 0.000002522 | 22.120 | 0.0152 | 0.0144 | 0.289 | 0.003 |
| rs282258 | T | C | 0.0993 | 0.0162 | 8.63E-10 | 37.563 | 0.0139 | 0.0094 | 0.138 | 0.005 |
| rs34291323 | T | C | 0.0954 | 0.0198 | 0.000001487 | 23.208 | -0.0063 | 0.0097 | 0.516 | 0.004 |
| rs41282644 | A | G | 0.1401 | 0.0303 | 0.000003737 | 21.374 | -0.046 | 0.0224 | 0.040 | 0.003 |
| rs6532374 | T | C | -0.1033 | 0.0226 | 0.000004613 | 20.887 | 0.0015 | 0.0122 | 0.903 | 0.003 |
| rs6921438 | A | G | -0.3204 | 0.0246 | 8.707E-39 | 169.536 | -0.0156 | 0.0093 | 0.094 | 0.047 |
| rs6993770 | A | T | 0.0918 | 0.0188 | 0.00000106 | 23.838 | 0.0121 | 0.0103 | 0.238 | 0.003 |
| rs71361173 | T | G | 0.1105 | 0.0238 | 0.000003572 | 21.551 | -0.0091 | 0.0134 | 0.497 | 0.003 |
| rs72831623 | A | G | 0.197 | 0.0369 | 9.29E-08 | 28.495 | -0.0079 | 0.0216 | 0.714 | 0.003 |
| rs782107 | A | G | 0.0765 | 0.0156 | 9.125E-07 | 24.042 | 0.0182 | 0.0093 | 0.052 | 0.003 |
| rs9472183 | A | G | -0.1006 | 0.0157 | 1.384E-10 | 41.048 | -0.0046 | 0.0097 | 0.631 | 0.005 |
| **IL-13** |  |  |  |  |  |  |  |  |  |  |
| rs10995615 | T | C | -0.1591 | 0.0341 | 0.000003118 | 21.757 | 0.0057 | 0.0118 | 0.630 | 0.006 |
| rs117795020 | A | G | -0.3584 | 0.0716 | 5.479E-07 | 25.042 | 0.0148 | 0.0344 | 0.668 | 0.007 |
| rs12623722 | A | G | -0.1189 | 0.0257 | 0.000003614 | 21.392 | -0.0006 | 0.0102 | 0.952 | 0.006 |
| rs138854806 | A | G | -0.4204 | 0.0839 | 5.449E-07 | 25.093 | 0.0327 | 0.0688 | 0.635 | 0.007 |
| rs139083458 | T | C | 0.9995 | 0.211 | 0.000002165 | 22.426 | -0.0285 | 0.0355 | 0.422 | 0.006 |
| rs147747784 | C | G | 0.3582 | 0.0754 | 0.000002039 | 22.557 | -0.0576 | 0.0269 | 0.032 | 0.006 |
| rs27949 | T | C | -0.1144 | 0.025 | 0.000004827 | 20.928 | 0.0115 | 0.01 | 0.249 | 0.006 |
| rs6799107 | T | C | -0.095 | 0.0206 | 0.000003992 | 21.262 | 0.0152 | 0.0114 | 0.182 | 0.003 |
| rs6921438 | A | G | -0.3204 | 0.0246 | 8.707E-39 | 169.536 | -0.0156 | 0.0093 | 0.094 | 0.047 |
| rs7073807 | T | C | 0.1618 | 0.0354 | 0.00000477 | 20.879 | 0.0137 | 0.0148 | 0.354 | 0.006 |
| rs75383097 | C | G | -0.5369 | 0.116 | 0.000003702 | 21.411 | -0.0277 | 0.0395 | 0.484 | 0.006 |
| rs76339001 | A | T | -0.4375 | 0.0886 | 7.915E-07 | 24.370 | 0.0089 | 0.0289 | 0.760 | 0.007 |
| rs77955971 | A | C | 0.4408 | 0.0868 | 3.756E-07 | 25.775 | 0.0352 | 0.0297 | 0.236 | 0.007 |
| **IL-16** |  |  |  |  |  |  |  |  |  |  |
| rs116135478 | A | G | 0.8296 | 0.1637 | 0.000000405 | 25.668 | -0.0473 | 0.0328 | 0.149 | 0.007 |
| rs117217798 | T | C | -0.2064 | 0.044 | 0.000002772 | 21.992 | 0.0052 | 0.0187 | 0.783 | 0.006 |
| rs117916513 | A | G | -0.4713 | 0.0982 | 0.000001605 | 23.021 | -0.0218 | 0.0419 | 0.603 | 0.006 |
| rs1255143 | T | C | 0.1387 | 0.0241 | 8.529E-09 | 33.103 | 0.0252 | 0.0093 | 0.007 | 0.009 |
| rs144691581 | A | G | 0.4929 | 0.0958 | 2.668E-07 | 26.457 | 0.054 | 0.0391 | 0.168 | 0.007 |
| rs1801020 | A | G | 0.1678 | 0.0271 | 5.628E-10 | 38.318 | -0.0016 | 0.0107 | 0.884 | 0.011 |
| rs4253283 | T | C | 0.1506 | 0.026 | 7.215E-09 | 33.532 | -0.0053 | 0.01 | 0.597 | 0.009 |
| rs4778636 | A | G | -0.7286 | 0.063 | 6.208E-31 | 133.675 | -0.0002 | 0.016 | 0.990 | 0.037 |
| rs9706053 | T | C | 0.4412 | 0.0928 | 0.000001976 | 22.591 | 0.0336 | 0.0332 | 0.312 | 0.006 |
| **IL-17** |  |  |  |  |  |  |  |  |  |  |
| rs117556572 | T | C | -0.5256 | 0.1097 | 0.00000166 | 22.943 | 0.0101 | 0.0352 | 0.774 | 0.006 |
| rs12735700 | T | G | -0.0943 | 0.0206 | 0.0000045 | 20.950 | 0.0148 | 0.0108 | 0.168 | 0.003 |
| rs149738638 | T | C | -0.1553 | 0.0337 | 0.000004106 | 21.231 | 0.0138 | 0.0181 | 0.445 | 0.003 |
| rs17282552 | T | C | -0.2026 | 0.0403 | 4.876E-07 | 25.267 | 0.0206 | 0.0261 | 0.431 | 0.003 |
| rs3804753 | A | G | 0.0943 | 0.0166 | 1.376E-08 | 32.262 | -0.0139 | 0.0094 | 0.140 | 0.004 |
| rs61990749 | C | G | 0.1124 | 0.0226 | 6.569E-07 | 24.729 | -0.02 | 0.0147 | 0.172 | 0.003 |
| rs78296352 | T | G | 0.3419 | 0.065 | 0.000000142 | 27.660 | 0.0109 | 0.024 | 0.649 | 0.004 |
| rs78629931 | T | C | 0.236 | 0.0471 | 5.462E-07 | 25.100 | 0.0151 | 0.0186 | 0.416 | 0.003 |
| **IL-18** |  |  |  |  |  |  |  |  |  |  |
| rs10414578 | T | C | -0.1817 | 0.0347 | 1.643E-07 | 27.404 | 0.0036 | 0.0155 | 0.814 | 0.007 |
| rs116383510 | A | C | -0.5412 | 0.1052 | 2.699E-07 | 26.451 | 0.0012 | 0.0406 | 0.976 | 0.007 |
| rs117266781 | T | C | 0.7051 | 0.1436 | 9.176E-07 | 24.097 | 0.0545 | 0.0424 | 0.199 | 0.007 |
| rs12420140 | A | G | -0.2479 | 0.0261 | 1.949E-21 | 90.165 | -0.005 | 0.0103 | 0.630 | 0.024 |
| rs143370787 | C | G | -0.3447 | 0.066 | 1.749E-07 | 27.262 | -0.003 | 0.0168 | 0.856 | 0.007 |
| rs17229943 | A | C | -0.3076 | 0.0463 | 3.062E-11 | 44.114 | 0.0023 | 0.0211 | 0.914 | 0.012 |
| rs1979967 | T | C | 0.14 | 0.0285 | 8.719E-07 | 24.117 | 0.0215 | 0.0111 | 0.052 | 0.007 |
| rs385076 | T | C | -0.2472 | 0.0247 | 1.559E-23 | 100.107 | 0.004 | 0.0098 | 0.685 | 0.027 |
| rs4482818 | A | G | 0.1233 | 0.0243 | 4.108E-07 | 25.732 | 0.0173 | 0.0096 | 0.072 | 0.007 |
| rs610473 | A | G | 0.1274 | 0.0242 | 1.433E-07 | 27.699 | 0.005 | 0.0097 | 0.609 | 0.007 |
| rs7444013 | A | G | -0.5318 | 0.0955 | 2.586E-08 | 30.992 | 0.0707 | 0.0405 | 0.081 | 0.008 |
| rs78623212 | T | C | 0.8322 | 0.1676 | 0.000000682 | 24.642 | 0.0376 | 0.0301 | 0.211 | 0.007 |
| rs78716465 | A | G | 0.3173 | 0.0679 | 0.000002981 | 21.825 | -0.0567 | 0.0242 | 0.019 | 0.006 |
| **IP-10** |  |  |  |  |  |  |  |  |  |  |
| rs113831257 | A | G | 0.3639 | 0.0641 | 1.388E-08 | 32.212 | 0.0228 | 0.0237 | 0.337 | 0.009 |
| rs143799975 | A | G | -0.7551 | 0.1638 | 0.000004012 | 21.240 | -0.0156 | 0.0427 | 0.714 | 0.006 |
| rs34383175 | T | C | -0.3196 | 0.0653 | 9.904E-07 | 23.942 | -0.0232 | 0.0251 | 0.355 | 0.006 |
| rs397816 | T | C | 0.1211 | 0.0248 | 0.000001026 | 23.832 | -0.0029 | 0.0094 | 0.759 | 0.006 |
| rs4862111 | T | C | 0.1448 | 0.0317 | 0.000004844 | 20.854 | 0.0012 | 0.0125 | 0.926 | 0.006 |
| rs6707974 | A | G | 0.1574 | 0.0337 | 0.000003027 | 21.803 | -0.0214 | 0.0127 | 0.091 | 0.006 |
| rs75970138 | A | G | -0.4845 | 0.1037 | 0.000002994 | 21.817 | -0.0221 | 0.0522 | 0.672 | 0.006 |
| rs7645625 | T | G | -0.1116 | 0.0236 | 0.000002192 | 22.350 | 0.0015 | 0.0094 | 0.871 | 0.006 |
| rs79848609 | A | C | 0.2514 | 0.0535 | 0.000002637 | 22.069 | -0.0284 | 0.0223 | 0.204 | 0.006 |
| rs8112909 | A | G | -0.139 | 0.0297 | 0.000002956 | 21.892 | 0.0025 | 0.0116 | 0.830 | 0.006 |
| rs9450351 | T | C | -0.2651 | 0.0488 | 5.475E-08 | 29.495 | 0.0029 | 0.0187 | 0.876 | 0.008 |
| **M-CSF** |  |  |  |  |  |  |  |  |  |  |
| rs116274860 | T | G | 0.8262 | 0.1739 | 0.000002029 | 22.554 | 0.0139 | 0.0368 | 0.706 | 0.009 |
| rs117867915 | T | C | 0.5224 | 0.1096 | 0.000001874 | 22.701 | 0.0017 | 0.0467 | 0.971 | 0.009 |
| rs11963606 | C | G | -0.5353 | 0.117 | 0.000004731 | 20.916 | -0.0013 | 0.0376 | 0.973 | 0.008 |
| rs12962919 | T | C | 0.3025 | 0.0659 | 0.000004394 | 21.054 | -0.0219 | 0.0154 | 0.154 | 0.008 |
| rs34089869 | T | C | 0.2194 | 0.0462 | 0.000002078 | 22.534 | 0.0339 | 0.0147 | 0.022 | 0.009 |
| rs4269021 | C | G | -0.2459 | 0.0504 | 0.000001051 | 23.785 | -0.0052 | 0.0134 | 0.699 | 0.009 |
| rs56367447 | T | C | -0.4878 | 0.0876 | 2.569E-08 | 30.983 | 0.0084 | 0.024 | 0.726 | 0.012 |
| rs62294910 | A | G | 0.3472 | 0.0687 | 4.378E-07 | 25.521 | 0.0112 | 0.0203 | 0.581 | 0.010 |
| rs72723242 | T | G | -0.4969 | 0.1083 | 0.000004434 | 21.035 | -0.0103 | 0.0202 | 0.610 | 0.008 |
| rs78296352 | T | G | 0.3419 | 0.065 | 0.000000142 | 27.660 | 0.0109 | 0.024 | 0.649 | 0.004 |
| rs9387100 | T | C | -0.135 | 0.029 | 0.000003341 | 21.653 | -0.0047 | 0.0098 | 0.628 | 0.009 |
| **MCAF** |  |  |  |  |  |  |  |  |  |  |
| rs10744620 | T | C | 0.0783 | 0.0161 | 0.000001118 | 23.647 | -0.0211 | 0.0096 | 0.028 | 0.003 |
| rs111995966 | T | G | 0.1428 | 0.0309 | 0.000003788 | 21.352 | -0.0248 | 0.0342 | 0.468 | 0.003 |
| rs12073356 | A | G | -0.1436 | 0.031 | 0.000003489 | 21.453 | 0.0033 | 0.0192 | 0.865 | 0.003 |
| rs12075 | A | G | 0.3724 | 0.0236 | 3.459E-56 | 248.857 | -0.0028 | 0.0094 | 0.768 | 0.066 |
| rs143815843 | A | G | -0.2049 | 0.0447 | 0.000004609 | 21.007 | 0.0518 | 0.0503 | 0.303 | 0.003 |
| rs146522229 | T | C | -0.5942 | 0.1161 | 3.093E-07 | 26.188 | 0.0243 | 0.043 | 0.572 | 0.003 |
| rs2036297 | A | G | 0.1182 | 0.016 | 1.302E-13 | 54.562 | -0.0292 | 0.0098 | 0.003 | 0.007 |
| rs2288370 | T | C | -0.1036 | 0.0162 | 1.555E-10 | 40.887 | -0.0068 | 0.0096 | 0.480 | 0.005 |
| rs56212190 | T | C | 0.1799 | 0.0372 | 0.000001318 | 23.381 | 0.0182 | 0.0226 | 0.420 | 0.003 |
| rs7033586 | A | G | -0.22 | 0.0467 | 0.000002426 | 22.187 | -0.0888 | 0.1006 | 0.378 | 0.003 |
| rs7197349 | A | G | 0.0971 | 0.0206 | 0.000002399 | 22.213 | -0.0144 | 0.014 | 0.304 | 0.003 |
| rs7517040 | A | G | -0.097 | 0.019 | 3.409E-07 | 26.057 | -0.0023 | 0.0112 | 0.838 | 0.003 |
| rs7632755 | A | G | 0.2984 | 0.0315 | 2.791E-21 | 89.717 | 0.0021 | 0.0183 | 0.909 | 0.011 |
| rs9317045 | A | C | 0.1157 | 0.0235 | 8.425E-07 | 24.234 | 0.027 | 0.0127 | 0.033 | 0.003 |
| **MCP-3** |  |  |  |  |  |  |  |  |  |  |
| rs10892381 | T | C | 0.2432 | 0.0473 | 2.693E-07 | 26.388 | -0.0097 | 0.0103 | 0.346 | 0.023 |
| rs117286643 | A | G | 0.6934 | 0.1474 | 0.000002542 | 22.089 | 0.048 | 0.0364 | 0.187 | 0.020 |
| rs2838065 | A | G | -0.221 | 0.0479 | 0.000003924 | 21.248 | 0.0037 | 0.0119 | 0.755 | 0.019 |
| rs28394764 | A | T | 0.597 | 0.1282 | 0.000003194 | 21.646 | -0.0014 | 0.0212 | 0.948 | 0.019 |
| rs3129806 | T | C | -0.1975 | 0.0433 | 0.000004978 | 20.767 | -0.01 | 0.0096 | 0.296 | 0.019 |
| rs62492260 | T | G | -0.2802 | 0.0578 | 0.000001229 | 23.458 | 0.0014 | 0.0138 | 0.922 | 0.021 |
| **MIF** |  |  |  |  |  |  |  |  |  |  |
| rs113218956 | A | G | -0.8789 | 0.1876 | 0.000002815 | 21.937 | 0.0405 | 0.0745 | 0.586 | 0.006 |
| rs11551183 | C | G | 0.3666 | 0.0795 | 0.000003999 | 21.252 | 0.0061 | 0.0248 | 0.805 | 0.006 |
| rs12594190 | A | G | 0.1321 | 0.0266 | 0.000000685 | 24.649 | 0.0165 | 0.0111 | 0.138 | 0.007 |
| rs13142904 | T | C | -0.2232 | 0.0425 | 1.468E-07 | 27.565 | -0.0064 | 0.0186 | 0.729 | 0.008 |
| rs141009259 | T | C | -0.6194 | 0.1285 | 0.000001444 | 23.221 | -0.0446 | 0.0441 | 0.312 | 0.007 |
| rs2294689 | C | G | -0.1338 | 0.0287 | 0.000003043 | 21.722 | 0.0315 | 0.0211 | 0.135 | 0.006 |
| rs2330634 | C | G | 0.1549 | 0.0249 | 4.571E-10 | 38.677 | -0.0022 | 0.0095 | 0.816 | 0.011 |
| rs35890933 | T | G | 0.1676 | 0.0365 | 0.000004458 | 21.073 | -0.0002 | 0.0116 | 0.983 | 0.006 |
| rs3814097 | A | G | -0.1163 | 0.0251 | 0.000003548 | 21.457 | 0.0046 | 0.0095 | 0.627 | 0.006 |
| rs78098071 | T | C | -0.4583 | 0.0915 | 5.509E-07 | 25.073 | -0.0337 | 0.0428 | 0.431 | 0.007 |
| **MIG** |  |  |  |  |  |  |  |  |  |  |
| rs111607343 | A | G | -0.5235 | 0.1119 | 0.000002928 | 21.875 | 0.0198 | 0.0268 | 0.460 | 0.006 |
| rs11177248 | A | G | 0.3157 | 0.0667 | 0.000002222 | 22.391 | 0.018 | 0.0189 | 0.340 | 0.006 |
| rs112861654 | A | G | -0.2682 | 0.0527 | 3.639E-07 | 25.886 | 0.0109 | 0.0174 | 0.529 | 0.007 |
| rs117831247 | T | C | -0.8819 | 0.173 | 3.445E-07 | 25.972 | 0.0778 | 0.0435 | 0.073 | 0.007 |
| rs13143163 | C | G | 0.2735 | 0.0582 | 0.000002622 | 22.072 | 0.0063 | 0.0198 | 0.753 | 0.006 |
| rs139010077 | T | C | 0.4337 | 0.0943 | 0.000004193 | 21.141 | -0.0039 | 0.0404 | 0.923 | 0.006 |
| rs1796086 | T | C | -0.2172 | 0.04 | 5.623E-08 | 29.469 | 0.0006 | 0.0167 | 0.973 | 0.008 |
| rs191555775 | A | T | 0.2279 | 0.0412 | 3.278E-08 | 30.582 | -0.0074 | 0.0159 | 0.641 | 0.008 |
| rs55876513 | T | G | 0.1638 | 0.0254 | 1.048E-10 | 41.565 | 0.0121 | 0.0114 | 0.287 | 0.011 |
| rs62562991 | A | G | 0.6239 | 0.1259 | 7.237E-07 | 24.544 | -0.0407 | 0.034 | 0.231 | 0.007 |
| rs6679677 | A | C | 0.1628 | 0.0327 | 6.514E-07 | 24.773 | 0.0086 | 0.0153 | 0.576 | 0.007 |
| rs77086208 | T | C | 0.327 | 0.0694 | 0.000002501 | 22.189 | 0.0321 | 0.0354 | 0.365 | 0.006 |
| rs816960 | T | C | -0.1179 | 0.0242 | 0.00000115 | 23.723 | 0.0043 | 0.0113 | 0.702 | 0.006 |
| **MIP-1A** |  |  |  |  |  |  |  |  |  |  |
| rs116615337 | A | G | 0.1286 | 0.0278 | 0.000003657 | 21.387 | 0.0008 | 0.0114 | 0.945 | 0.006 |
| rs12690897 | A | G | 0.1215 | 0.026 | 0.000003073 | 21.825 | 0.0093 | 0.0107 | 0.385 | 0.006 |
| rs184154340 | A | G | 0.3251 | 0.0689 | 0.000002399 | 22.251 | 0.0212 | 0.0239 | 0.376 | 0.006 |
| rs57786342 | A | G | 0.139 | 0.0283 | 8.909E-07 | 24.111 | 0.0123 | 0.0115 | 0.286 | 0.007 |
| rs60198979 | A | G | -0.2154 | 0.0455 | 0.000002215 | 22.399 | 0.005 | 0.017 | 0.767 | 0.006 |
| rs6900267 | A | C | -0.2472 | 0.0515 | 0.000001604 | 23.027 | 0.001 | 0.0216 | 0.961 | 0.006 |
| **MIP-1B** |  |  |  |  |  |  |  |  |  |  |
| rs113010081 | T | C | -0.5799 | 0.0236 | 1.57E-133 | 603.639 | -0.0223 | 0.0146 | 0.128 | 0.068 |
| rs113877493 | T | C | -0.607 | 0.0217 | 3.67E-172 | 782.264 | 0.0003 | 0.0171 | 0.985 | 0.086 |
| rs116237296 | A | G | 0.5284 | 0.1115 | 0.000002153 | 22.453 | 0.0156 | 0.0444 | 0.726 | 0.003 |
| rs117453826 | A | G | -0.5907 | 0.0591 | 1.53E-23 | 99.874 | 0.0218 | 0.0367 | 0.552 | 0.012 |
| rs117657747 | A | G | 0.2089 | 0.0453 | 0.000004013 | 21.259 | 0.0099 | 0.0192 | 0.606 | 0.003 |
| rs141102180 | T | G | 0.3298 | 0.0392 | 3.751E-17 | 70.766 | 0.0237 | 0.0359 | 0.508 | 0.008 |
| rs1437220 | T | C | 0.1437 | 0.0315 | 0.000004916 | 20.806 | -0.0133 | 0.0208 | 0.524 | 0.003 |
| rs17138331 | A | G | -0.1434 | 0.0295 | 0.000001125 | 23.624 | 0.0095 | 0.0144 | 0.509 | 0.003 |
| rs2411161 | T | C | 0.1719 | 0.0365 | 0.000002547 | 22.175 | -0.0071 | 0.0204 | 0.727 | 0.003 |
| rs281748 | C | G | -0.0794 | 0.0171 | 0.000003277 | 21.555 | 0.0051 | 0.01 | 0.610 | 0.003 |
| rs3760440 | A | G | 0.1242 | 0.0162 | 1.734E-14 | 58.764 | 0.0167 | 0.0098 | 0.088 | 0.007 |
| rs5743614 | T | C | 0.1115 | 0.0232 | 0.000001569 | 23.092 | -0.0134 | 0.0122 | 0.276 | 0.003 |
| rs6908843 | A | G | 0.0997 | 0.0209 | 0.000001779 | 22.751 | 0.0315 | 0.012 | 0.009 | 0.003 |
| rs72791296 | T | C | 0.2364 | 0.0466 | 3.968E-07 | 25.729 | 0.0215 | 0.0224 | 0.339 | 0.003 |
| rs72799710 | T | C | -0.1037 | 0.0217 | 0.000001792 | 22.831 | 0.0089 | 0.012 | 0.459 | 0.003 |
| rs76356863 | A | T | -0.3456 | 0.0667 | 2.219E-07 | 26.841 | -0.0179 | 0.0273 | 0.512 | 0.003 |
| rs76582507 | A | G | 0.3259 | 0.0676 | 0.000001421 | 23.236 | 0.0647 | 0.0365 | 0.076 | 0.003 |
| rs76776296 | A | G | 0.313 | 0.0598 | 1.632E-07 | 27.389 | -0.0126 | 0.0242 | 0.603 | 0.003 |
| rs79068918 | C | G | 0.2674 | 0.0271 | 5.537E-23 | 97.337 | 0.0097 | 0.0156 | 0.535 | 0.012 |
| rs9793308 | A | G | 0.0842 | 0.0177 | 0.000002042 | 22.624 | -0.0001 | 0.0115 | 0.992 | 0.003 |
| **PDGF-BB** |  |  |  |  |  |  |  |  |  |  |
| rs11247305 | C | G | -0.1687 | 0.0364 | 0.000003468 | 21.475 | 0.0041 | 0.025 | 0.871 | 0.003 |
| rs116445074 | T | G | 0.2869 | 0.0587 | 0.000001017 | 23.883 | 0.0123 | 0.0388 | 0.750 | 0.003 |
| rs11766649 | A | G | 0.0902 | 0.0196 | 0.000003964 | 21.174 | 0.0001 | 0.0106 | 0.996 | 0.003 |
| rs12289510 | A | G | -0.0772 | 0.0158 | 0.000001001 | 23.868 | 0.0049 | 0.0093 | 0.601 | 0.003 |
| rs13037046 | A | T | -0.0948 | 0.0206 | 0.000003958 | 21.173 | 0.0064 | 0.011 | 0.559 | 0.003 |
| rs13412535 | A | G | -0.1043 | 0.0213 | 9.671E-07 | 23.972 | -0.0111 | 0.011 | 0.314 | 0.003 |
| rs2324229 | T | C | 0.0884 | 0.0161 | 4.016E-08 | 30.140 | 0.0158 | 0.0096 | 0.100 | 0.004 |
| rs35859699 | A | G | -0.3854 | 0.0838 | 0.000004223 | 21.146 | 0.0029 | 0.0312 | 0.927 | 0.003 |
| rs4965869 | T | C | 0.1843 | 0.0181 | 2.22E-24 | 103.655 | -0.0102 | 0.0105 | 0.331 | 0.012 |
| rs55680718 | T | C | -0.1359 | 0.0245 | 2.956E-08 | 30.761 | 0.0168 | 0.0147 | 0.253 | 0.004 |
| rs72777070 | T | G | -0.1048 | 0.02 | 1.557E-07 | 27.451 | 0.0122 | 0.012 | 0.307 | 0.003 |
| rs73162807 | A | C | -0.2313 | 0.0499 | 0.000003548 | 21.481 | -0.015 | 0.0319 | 0.639 | 0.003 |
| rs9936075 | A | G | -0.0767 | 0.0163 | 0.000002675 | 22.137 | 0.0179 | 0.0098 | 0.068 | 0.003 |
| rs9941733 | A | G | 0.1156 | 0.0229 | 4.331E-07 | 25.476 | 0.0072 | 0.0124 | 0.562 | 0.003 |
| **RANTES** |  |  |  |  |  |  |  |  |  |  |
| rs112072646 | A | G | 0.4209 | 0.0859 | 9.617E-07 | 23.995 | 0.0523 | 0.0282 | 0.063 | 0.007 |
| rs147509526 | T | C | -0.3558 | 0.0715 | 6.567E-07 | 24.748 | 0.0233 | 0.0382 | 0.542 | 0.007 |
| rs2251660 | A | C | 0.1831 | 0.0356 | 2.691E-07 | 26.438 | 0.0126 | 0.013 | 0.334 | 0.008 |
| rs2731672 | T | C | -0.1242 | 0.0272 | 0.000004827 | 20.838 | -0.0026 | 0.0106 | 0.806 | 0.006 |
| rs62438851 | A | G | -0.1904 | 0.0413 | 0.000004009 | 21.241 | -0.0016 | 0.0136 | 0.908 | 0.006 |
| rs7000423 | T | C | -0.1314 | 0.0252 | 1.852E-07 | 27.173 | 0.0082 | 0.0099 | 0.409 | 0.008 |
| rs7170339 | C | G | -0.4283 | 0.0904 | 0.000002187 | 22.434 | -0.0122 | 0.0342 | 0.722 | 0.006 |
| rs72793342 | A | G | -0.1505 | 0.0307 | 0.000000908 | 24.018 | -0.0216 | 0.0116 | 0.064 | 0.007 |
| rs74472919 | T | C | 0.3547 | 0.06 | 3.353E-09 | 34.928 | -0.0011 | 0.0264 | 0.966 | 0.010 |
| rs9675798 | T | G | -0.2583 | 0.0552 | 0.000002888 | 21.884 | -0.0054 | 0.0246 | 0.827 | 0.006 |
| **SCF** |  |  |  |  |  |  |  |  |  |  |
| rs113127926 | A | C | 0.1974 | 0.0418 | 0.000002337 | 22.297 | -0.0143 | 0.0185 | 0.442 | 0.003 |
| rs13412535 | A | G | -0.1043 | 0.0213 | 9.671E-07 | 23.972 | -0.0111 | 0.011 | 0.314 | 0.003 |
| rs1557570 | T | G | 0.1172 | 0.0169 | 4.133E-12 | 48.081 | -0.0001 | 0.0099 | 0.992 | 0.006 |
| rs1568119 | T | C | -0.5946 | 0.1129 | 1.374E-07 | 27.730 | -0.0311 | 0.049 | 0.526 | 0.003 |
| rs4841899 | T | C | -0.1002 | 0.0178 | 1.673E-08 | 31.680 | -0.011 | 0.0099 | 0.269 | 0.004 |
| rs635634 | T | C | -0.1035 | 0.0191 | 5.705E-08 | 29.357 | 0.0339 | 0.012 | 0.005 | 0.004 |
| rs7039247 | C | G | 0.079 | 0.0168 | 0.00000246 | 22.107 | 0.0002 | 0.0099 | 0.984 | 0.003 |
| rs72678285 | A | T | 0.1062 | 0.0231 | 0.00000443 | 21.131 | -0.0067 | 0.0128 | 0.598 | 0.003 |
| rs78369473 | T | C | -0.2256 | 0.0484 | 0.00000314 | 21.721 | 0.0289 | 0.0274 | 0.291 | 0.003 |
| rs78666213 | T | G | -0.2845 | 0.0574 | 7.152E-07 | 24.560 | -0.0006 | 0.0263 | 0.982 | 0.003 |
| rs80271436 | A | G | -0.2393 | 0.0484 | 7.488E-07 | 24.439 | -0.0061 | 0.0215 | 0.776 | 0.003 |
| **SCGF-B** |  |  |  |  |  |  |  |  |  |  |
| rs112346514 | T | C | -0.3261 | 0.0703 | 0.000003543 | 21.506 | 0.0335 | 0.0277 | 0.225 | 0.006 |
| rs1149926 | T | C | -0.3458 | 0.0749 | 0.000003917 | 21.304 | 0.0055 | 0.0288 | 0.848 | 0.006 |
| rs116924815 | T | C | 0.6046 | 0.0737 | 2.251E-16 | 67.262 | -0.0044 | 0.0277 | 0.874 | 0.018 |
| rs117716477 | A | C | 0.8242 | 0.084 | 1.029E-22 | 96.222 | 0.0497 | 0.0394 | 0.208 | 0.025 |
| rs12480722 | T | C | 0.1654 | 0.0353 | 0.000002812 | 21.943 | -0.0263 | 0.0146 | 0.072 | 0.006 |
| rs13287050 | A | T | -0.121 | 0.0263 | 0.000004118 | 21.156 | -0.0281 | 0.0099 | 0.005 | 0.006 |
| rs139413256 | A | G | -0.5174 | 0.1076 | 0.000001532 | 23.110 | 0.0101 | 0.0244 | 0.680 | 0.006 |
| rs143829871 | T | C | -0.1866 | 0.0399 | 0.000002852 | 21.860 | -0.035 | 0.0192 | 0.068 | 0.006 |
| rs149009264 | A | G | 0.4551 | 0.0985 | 0.000003793 | 21.336 | 0.0271 | 0.0413 | 0.512 | 0.006 |
| rs150733161 | T | C | -0.5255 | 0.112 | 0.000002687 | 22.003 | -0.0145 | 0.0394 | 0.713 | 0.006 |
| rs151194174 | A | G | 0.4536 | 0.0941 | 0.000001454 | 23.224 | -0.0033 | 0.0326 | 0.919 | 0.006 |
| rs17876031 | A | G | -0.1496 | 0.0254 | 3.667E-09 | 34.671 | 0.0039 | 0.0099 | 0.694 | 0.009 |
| rs264157 | A | G | 0.1079 | 0.0233 | 0.000003685 | 21.434 | -0.0042 | 0.0094 | 0.653 | 0.006 |
| rs34911860 | A | G | -0.3674 | 0.0787 | 0.000003002 | 21.782 | 0.0272 | 0.0478 | 0.570 | 0.006 |
| rs4656185 | A | G | 0.2103 | 0.0254 | 1.287E-16 | 68.514 | 0.0016 | 0.0099 | 0.868 | 0.018 |
| rs77954165 | T | C | 0.2631 | 0.0562 | 0.000002867 | 21.905 | 0.0095 | 0.0161 | 0.556 | 0.006 |
| rs7815967 | T | C | 0.1325 | 0.0288 | 0.000004371 | 21.155 | -0.001 | 0.0144 | 0.946 | 0.006 |
| rs78217154 | T | C | 0.3942 | 0.0861 | 0.000004722 | 20.950 | -0.0415 | 0.0325 | 0.202 | 0.006 |
| **SDF-1A** |  |  |  |  |  |  |  |  |  |  |
| rs10013755 | A | T | 0.5188 | 0.0995 | 1.851E-07 | 27.180 | -0.0175 | 0.0341 | 0.608 | 0.003 |
| rs10474392 | A | G | 0.0934 | 0.0177 | 1.376E-07 | 27.838 | -0.0036 | 0.011 | 0.748 | 0.004 |
| rs12141941 | T | C | -0.0881 | 0.0186 | 0.000002263 | 22.429 | -0.0008 | 0.0109 | 0.940 | 0.003 |
| rs149893336 | A | G | -0.494 | 0.1082 | 0.00000493 | 20.840 | -0.02 | 0.0349 | 0.567 | 0.003 |
| rs1600396 | A | G | -0.0933 | 0.0204 | 0.000004939 | 20.912 | 0.0002 | 0.0107 | 0.988 | 0.003 |
| rs3988298 | T | C | -0.1263 | 0.0266 | 0.000002124 | 22.539 | 0.0138 | 0.0142 | 0.329 | 0.003 |
| rs62194947 | T | C | -0.0852 | 0.0185 | 0.000004269 | 21.204 | 0.002 | 0.0105 | 0.846 | 0.003 |
| rs78037609 | A | G | -0.6261 | 0.1334 | 0.000002666 | 22.022 | 0.0102 | 0.0543 | 0.851 | 0.003 |
| rs78883416 | C | G | -0.0871 | 0.0182 | 0.000001755 | 22.897 | -0.0001 | 0.0099 | 0.995 | 0.003 |
| **TNF-A** |  |  |  |  |  |  |  |  |  |  |
| rs10834997 | A | G | -0.123 | 0.0256 | 0.000001528 | 23.072 | 0.0061 | 0.0101 | 0.543 | 0.007 |
| rs111332265 | A | G | -0.3678 | 0.0745 | 7.913E-07 | 24.359 | 0.0166 | 0.0203 | 0.414 | 0.007 |
| rs115669577 | A | G | 0.981 | 0.1994 | 8.625E-07 | 24.190 | -0.0263 | 0.0488 | 0.590 | 0.007 |
| rs79105320 | A | G | 0.5573 | 0.1177 | 0.000002207 | 22.407 | -0.0264 | 0.0359 | 0.462 | 0.006 |
| **TNF-B** |  |  |  |  |  |  |  |  |  |  |
| rs10925040 | T | C | 0.1738 | 0.0372 | 0.000002929 | 21.800 | -0.018 | 0.0096 | 0.062 | 0.014 |
| rs75240021 | C | G | 0.3713 | 0.0772 | 0.000001489 | 23.103 | -0.0332 | 0.0176 | 0.060 | 0.015 |
| rs753274 | T | C | -0.1725 | 0.037 | 0.000003143 | 21.708 | -0.0042 | 0.0093 | 0.653 | 0.014 |
| rs7629875 | A | G | 0.3841 | 0.0774 | 6.897E-07 | 24.595 | -0.004 | 0.0212 | 0.851 | 0.015 |
| rs78296352 | T | G | 0.3419 | 0.065 | 0.000000142 | 27.660 | 0.0109 | 0.024 | 0.649 | 0.004 |
| **TRAIL** |  |  |  |  |  |  |  |  |  |  |
| rs13278062 | T | G | 0.08 | 0.0157 | 3.326E-07 | 25.958 | -0.0048 | 0.0093 | 0.608 | 0.003 |
| rs138987090 | A | G | -0.7264 | 0.0749 | 2.974E-22 | 94.033 | 0.0507 | 0.0569 | 0.372 | 0.011 |
| rs148051545 | T | C | -0.4211 | 0.0843 | 5.843E-07 | 24.947 | -0.0226 | 0.0297 | 0.447 | 0.003 |
| rs17434886 | T | C | -0.0918 | 0.0199 | 0.000004195 | 21.275 | -0.0108 | 0.0127 | 0.394 | 0.003 |
| rs193112415 | T | C | -1.0456 | 0.062 | 1.008E-63 | 284.343 | -0.0792 | 0.0339 | 0.019 | 0.033 |
| rs28431810 | C | G | -0.1216 | 0.0252 | 0.000001406 | 23.279 | -0.0602 | 0.0509 | 0.237 | 0.003 |
| rs28521641 | A | T | -0.7004 | 0.0445 | 7.786E-56 | 247.666 | -0.0156 | 0.0257 | 0.545 | 0.029 |
| rs550057 | T | C | -0.0783 | 0.0169 | 0.000003707 | 21.461 | 0.0234 | 0.0107 | 0.028 | 0.003 |
| rs57396456 | T | C | -0.5641 | 0.0516 | 7.71E-28 | 119.483 | -0.0441 | 0.0266 | 0.098 | 0.014 |
| rs62093514 | T | C | 1.0459 | 0.0549 | 5.798E-81 | 362.852 | -0.0019 | 0.0288 | 0.947 | 0.042 |
| rs72899452 | T | C | 0.1223 | 0.0264 | 0.000003748 | 21.456 | 0.0136 | 0.0187 | 0.468 | 0.003 |
| rs73039026 | A | C | -0.3098 | 0.0634 | 0.000001015 | 23.871 | 0.0227 | 0.0432 | 0.599 | 0.003 |
| rs747324 | T | C | -0.0826 | 0.0178 | 0.000003338 | 21.529 | 0.0016 | 0.01 | 0.874 | 0.003 |
| rs74778900 | T | C | 0.5791 | 0.0531 | 9.901E-28 | 118.908 | -0.0453 | 0.0387 | 0.242 | 0.014 |
| rs75928541 | A | G | 0.2784 | 0.0591 | 0.000002442 | 22.185 | -0.028 | 0.0335 | 0.404 | 0.003 |
| rs79287178 | A | G | -0.4304 | 0.042 | 1.173E-24 | 104.988 | 0.0082 | 0.028 | 0.771 | 0.013 |
| **VEGF** |  |  |  |  |  |  |  |  |  |  |
| rs10411345 | C | G | -0.1041 | 0.0218 | 0.000001733 | 22.796 | -0.0073 | 0.0122 | 0.549 | 0.003 |
| rs10761731 | A | T | -0.0813 | 0.0167 | 0.000001068 | 23.694 | 0.0018 | 0.0094 | 0.850 | 0.003 |
| rs10934631 | T | C | -0.1132 | 0.0244 | 0.000003607 | 21.518 | 0.027 | 0.0116 | 0.020 | 0.003 |
| rs10967186 | T | C | 0.0899 | 0.0169 | 1.086E-07 | 28.289 | 0.0237 | 0.0093 | 0.011 | 0.004 |
| rs12456390 | T | C | -0.0818 | 0.0179 | 0.000004882 | 20.878 | 0.0147 | 0.0099 | 0.140 | 0.003 |
| rs13209117 | A | G | 0.0981 | 0.0186 | 1.271E-07 | 27.810 | 0.0023 | 0.0105 | 0.824 | 0.003 |
| rs143479231 | A | G | -0.2628 | 0.0489 | 7.902E-08 | 28.874 | -0.0437 | 0.0349 | 0.211 | 0.004 |
| rs3108686 | A | C | -0.7967 | 0.1702 | 0.000002861 | 21.903 | -0.0073 | 0.0389 | 0.851 | 0.004 |
| rs4082730 | A | G | 0.2455 | 0.0533 | 0.000004116 | 21.209 | 0.0359 | 0.0253 | 0.156 | 0.003 |
| rs6921438 | A | G | -0.3204 | 0.0246 | 8.707E-39 | 169.536 | -0.0156 | 0.0093 | 0.094 | 0.047 |
| rs7030781 | A | T | 0.1403 | 0.0172 | 3.454E-16 | 66.518 | 0.0195 | 0.0095 | 0.040 | 0.009 |
| rs73418461 | A | G | -0.2498 | 0.0521 | 0.00000161 | 22.982 | 0.0005 | 0.0206 | 0.980 | 0.003 |
| rs73872715 | T | C | -0.6079 | 0.1299 | 0.000002864 | 21.894 | 0.0183 | 0.0392 | 0.641 | 0.003 |
| rs8045833 | A | G | 0.103 | 0.0211 | 0.000001006 | 23.823 | -0.0049 | 0.0105 | 0.640 | 0.003 |
| rs9472183 | A | G | -0.1006 | 0.0157 | 1.384E-10 | 41.048 | -0.0046 | 0.0097 | 0.631 | 0.005 |

Abbreviations: se: standard error

**Table S4. MR estimates of KOA on forty-one inflammatory cytokines.**

| N | EXPOSURES | nSNP | MR Egger | | Weighted median | | Inverse variance weighted | | Simple mode | | Weighted mode | |
| --- | --- | --- | --- | --- | --- | --- | --- | --- | --- | --- | --- | --- |
|  |  |  | *P* | OR (95%CI) | *P* | OR (95%CI) | *P* | OR (95%CI) | *P* | OR (95%CI) | *P* | OR (95%CI) |
| 1 | B-NGF | 6 | 0.58 | 0.63 (0.14,2.86) | 0.10 | 0.73 (0.51,1.06) | 0.08 | 0.77 (0.57,1.03) | 0.24 | 0.67 (0.38,1.21) | 0.17 | 0.68 (0.43,1.09) |
| 2 | CTACK | 6 | 0.37 | 2.24 (0.47,10.65) | 0.60 | 0.90 (0.62, 1.32) | 0.55 | 0.91 (0.66, 1.25) | 0.55 | 0.82 (0.45, 1.50) | 0.60 | 1.15 (0.71, 1.86) |
| 3 | EOTAXIN | 6 | 0.49 | 1.47 (0.54,3.99) | 0.49 | 1.09 (0.85,1.41) | 0.51 | 1.07 (0.88,1.30) | 0.73 | 1.06 (0.76,1.49) | 0.54 | 1.10 (0.82,1.48) |
| 4 | FGF-BASIC | 6 | 0.60 | 0.74 (0.26,2.09) | 0.43 | 0.90 (0.70,1.16) | 0.85 | 0.98 (0.80,1.20) | 0.58 | 0.90 (0.62,1.29) | 0.52 | 0.89 (0.65,1.23) |
| 5 | G-CSF | 6 | 0.67 | 0.79 (0.29,2.19) | 0.65 | 1.06 (0.84,1.33) | 0.90 | 1.01 (0.83,1.24) | 0.63 | 1.09 (0.77,1.55) | 0.59 | 1.09 (0.81,1.47) |
| 6 | GROA | 6 | 0.19 | 3.40 (0.75,15.41) | 0.74 | 1.07 (0.73, 1.56) | 0.71 | 1.06 (0.79, 1.43) | 0.88 | 1.05 (0.60, 1.83) | 0.87 | 1.04 (0.64, 1.70) |
| 7 | HGF | 6 | 0.93 | 0.95 (0.30,2.97) | 0.86 | 0.98 (0.76,1.26) | 0.96 | 1.01 (0.82,1.23) | 0.91 | 0.98 (0.69,1.40) | 0.91 | 0.98 (0.73,1.33) |
| 8 | IFN-G | 6 | 0.48 | 1.59 (0.49,5.14) | 0.36 | 1.13 (0.87,1.47) | 0.49 | 1.08 (0.87,1.34) | 0.55 | 1.13 (0.77,1.65) | 0.43 | 1.15 (0.83,1.60) |
| 9 | IL-1B | 6 | 0.67 | 0.69 (0.14,3.32) | 0.88 | 0.97 (0.68,1.39) | 0.95 | 1.01 (0.74,1.37) | 0.98 | 0.99 (0.60,1.64) | 0.82 | 0.94 (0.59,1.50) |
| 10 | IL-1RA | 6 | 0.83 | 0.84 (0.19,3.70) | 0.45 | 0.87 (0.60,1.25) | 0.28 | 0.85 (0.63,1.14) | 0.64 | 0.88 (0.52,1.48) | 0.56 | 0.87 (0.55,1.36) |
| 11 | IL-2 | 6 | 0.98 | 1.02 (0.22,4.64) | 0.97 | 1.01 (0.71,1.44) | 0.54 | 0.91 (0.68,1.23) | 0.88 | 1.04 (0.61,1.79) | 0.89 | 1.04 (0.64,1.67) |
| 12 | IL-2RA | 6 | 0.96 | 0.95 (0.10,8.61) | 0.51 | 0.88 (0.60,1.29) | 0.88 | 0.97 (0.66,1.43) | 0.78 | 0.92 (0.54,1.58) | 0.65 | 0.90 (0.59,1.37) |
| 13 | IL-4 | 6 | 0.73 | 1.21 (0.44,3.30) | 0.21 | 1.17 (0.92,1.50) | 0.15 | 1.16 (0.95,1.41) | 0.39 | 1.19 (0.83,1.72) | 0.36 | 1.19 (0.85,1.66) |
| 14 | IL-5 | 6 | 0.83 | 1.30 (0.15,11.43) | 0.47 | 0.86 (0.57, 1.30) | 0.12 | 0.73 (0.49, 1.09) | 0.84 | 0.92 (0.45, 1.89) | 0.77 | 0.92 (0.54, 1.56) |
| 15 | IL-6 | 6 | 0.59 | 1.34 (0.49,3.65) | 0.56 | 1.08 (0.84,1.38) | 0.60 | 1.05 (0.87,1.28) | 0.48 | 1.16 (0.79,1.69) | 0.52 | 1.12 (0.82,1.54) |
| 16 | IL-7 | 6 | 0.80 | 0.81 (0.17,3.74) | 0.48 | 0.87 (0.60,1.27) | 0.12 | 0.79 (0.58,1.06) | 0.95 | 0.98 (0.57,1.70) | 0.77 | 0.92 (0.56,1.53) |
| 17 | IL-8 | 6 | 0.56 | 0.61 (0.14,2.77) | 0.85 | 1.03 (0.72,1.48) | 0.86 | 0.97 (0.72,1.31) | 0.66 | 1.12 (0.69,1.82) | 0.65 | 1.12 (0.71,1.78) |
| 18 | IL-9 | 6 | 0.80 | 1.23 (0.28,5.45) | 0.68 | 1.08 (0.76,1.52) | 0.90 | 0.98 (0.73,1.32) | 0.78 | 1.08 (0.65,1.79) | 0.77 | 1.08 (0.68,1.72) |
| 19 | IL-10 | 6 | 0.31 | 1.85 (0.66,5.18) | 0.36 | 0.89 (0.69,1.14) | 0.11 | 0.85 (0.69,1.04) | 0.60 | 0.89 (0.60,1.33) | 0.63 | 0.92 (0.66,1.27) |
| 20 | IL-12-P70 | 6 | 0.63 | 1.30 (0.48,3.52) | 0.60 | 0.93 (0.73,1.20) | 0.23 | 0.89 (0.73,1.08) | 0.67 | 0.92 (0.65,1.31) | 0.68 | 0.94 (0.70,1.25) |
| 21 | IL-13 | 6 | 0.91 | 1.12 (0.17,7.22) | 0.37 | 0.85 (0.58,1.22) | 0.17 | 0.79 (0.57,1.10) | 0.55 | 0.85 (0.51,1.41) | 0.50 | 0.85 (0.54,1.32) |
| 22 | IL-16 | 6 | 0.76 | 1.28 (0.28,5.82) | 0.89 | 1.03 (0.71,1.48) | 0.90 | 0.98 (0.73,1.32) | 0.69 | 1.12 (0.66,1.91) | 0.85 | 1.05 (0.65,1.70) |
| 23 | IL-17 | 3 | 0.69 | 3.63 (0.03,437.08) | 0.64 | 1.10 (0.75, 1.60) | 0.76 | 1.05 (0.76, 1.45) | 0.65 | 1.12 (0.73, 1.70) | 0.66 | 1.12 (0.73, 1.73) |
| 24 | IL-18 | 6 | 0.73 | 0.72 (0.12,4.30) | 0.19 | 1.29 (0.88,1.90) | 0.10 | 1.33 (0.95,1.86) | 0.38 | 1.35 (0.73,2.50) | 0.34 | 1.30 (0.79,2.13) |
| 25 | IP-10 | 6 | 0.48 | 1.79 (0.41,7.84) | 0.80 | 1.05 (0.74,1.49) | 0.70 | 0.95 (0.71,1.26) | 0.85 | 1.06 (0.61,1.82) | 0.67 | 1.11 (0.71,1.73) |
| 26 | M-CSF | 6 | 0.77 | 1.40 (0.17,11.55) | 0.87 | 0.96 (0.60, 1.54) | 0.75 | 0.94 (0.64, 1.37) | 0.85 | 1.08 (0.52, 2.24) | 0.99 | 0.99 (0.55, 1.81) |
| 27 | MCP-1-MCAF | 6 | 0.69 | 1.32 (0.36,4.80) | 0.92 | 1.01 (0.78,1.31) | 0.90 | 1.01 (0.80,1.28) | 0.96 | 0.99 (0.68,1.44) | 0.93 | 1.02 (0.74,1.39) |
| 28 | MCP-3 | 6 | 0.76 | 1.61 (0.09,27.44) | 0.31 | 1.39 (0.74, 2.63) | 0.18 | 1.44 (0.85, 2.45) | 0.18 | 2.16 (0.82, 5.70) | 0.39 | 1.49 (0.65, 3.45) |
| 29 | MIF | 6 | 0.73 | 0.70 (0.11,4.56) | 0.36 | 0.84 (0.57,1.23) | 0.68 | 0.93 (0.67,1.30) | 0.46 | 0.78 (0.42,1.45) | 0.32 | 0.76 (0.47,1.24) |
| 30 | MIG | 6 | 0.58 | 0.64 (0.15,2.78) | 0.39 | 0.86 (0.61,1.21) | 0.45 | 0.89 (0.67,1.20) | 0.61 | 0.87 (0.53,1.44) | 0.49 | 0.84 (0.53,1.33) |
| 31 | MIP-1A | 6 | 0.82 | 0.83 (0.18,3.75) | 0.06 | 0.70 (0.49,1.02) | 0.03 | 0.72 (0.54,0.97) | 0.22 | 0.70 (0.42,1.15) | 0.20 | 0.70 (0.44,1.12) |
| 32 | MIP-1B | 6 | 0.37 | 0.60 (0.22,1.62) | 0.77 | 0.96 (0.76,1.23) | 0.59 | 1.06 (0.87,1.28) | 0.84 | 0.96 (0.66,1.40) | 0.71 | 0.94 (0.69,1.28) |
| 33 | PDGF-BB | 6 | 0.21 | 0.47 (0.17,1.27) | 0.98 | 1.00 (0.79,1.26) | 0.63 | 1.05 (0.86,1.28) | 0.27 | 1.28 (0.86,1.91) | 0.61 | 0.92 (0.68,1.24) |
| 34 | RANTES | 6 | 0.98 | 1.02 (0.15,7.12) | 0.87 | 0.97 (0.65,1.43) | 0.68 | 1.08 (0.76,1.52) | 0.66 | 0.87 (0.49,1.56) | 0.73 | 0.92 (0.57,1.46) |
| 35 | SCF | 6 | 0.64 | 0.74 (0.23,2.41) | 0.37 | 1.12 (0.87,1.43) | 0.83 | 1.02 (0.82,1.27) | 0.29 | 1.26 (0.86,1.84) | 0.25 | 1.25 (0.89,1.76) |
| 36 | SCGF-B | 6 | 0.22 | 0.34 (0.08,1.47) | 0.29 | 0.82 (0.57,1.18) | 0.20 | 0.83 (0.62,1.10) | 0.52 | 0.82 (0.46,1.44) | 0.31 | 0.76 (0.48,1.22) |
| 37 | SDF-1A | 6 | 0.91 | 1.07 (0.34,3.40) | 0.59 | 1.08 (0.82,1.41) | 0.78 | 0.97 (0.79,1.19) | 0.56 | 1.12 (0.78,1.61) | 0.58 | 1.10 (0.80,1.52) |
| 38 | TNF-A | 6 | 0.90 | 1.11 (0.24,5.11) | 0.78 | 1.06 (0.71,1.56) | 0.64 | 1.07 (0.80,1.45) | 0.83 | 1.06 (0.63,1.80) | 0.81 | 1.06 (0.67,1.67) |
| 39 | TNF-B | 6 | 0.61 | 1.87 (0.20,17.49) | 0.65 | 1.14 (0.65, 1.97) | 0.90 | 1.03 (0.66, 1.61) | 0.72 | 1.16 (0.55, 2.43) | 0.67 | 1.17 (0.60, 2.28) |
| 40 | TRAIL | 6 | 0.69 | 0.81 (0.30,2.18) | 0.41 | 0.91 (0.71,1.15) | 0.74 | 0.97 (0.79,1.18) | 0.44 | 0.86 (0.61,1.22) | 0.44 | 0.87 (0.64,1.20) |
| 41 | VEGF | 6 | 0.27 | 2.21 (0.65,7.53) | 0.15 | 1.23 (0.93,1.63) | 0.23 | 1.16 (0.91,1.48) | 0.65 | 1.12 (0.71,1.76) | 0.15 | 1.35 (0.95,1.92) |

**Table S5. Heterogeneity and horizontal pleiotropy tests of KOA on forty-one inflammatory cytokines.**

| N | Exposures | Q1 pval | Q2 pval | intercept | intercept pval | MR-PRESSO pval | Recommended Method |
| --- | --- | --- | --- | --- | --- | --- | --- |
| 1 | B-NGF | 0.567 | 0.432 | 0.013 | 0.810 | 0.61 | IVW |
| 2 | CTACK | 0.319 | 0.355 | -0.062 | 0.310 | 0.32 | IVW |
| 3 | EOTAXIN | 0.810 | 0.760 | -0.022 | 0.560 | 0.85 | IVW |
| 4 | FGF-BASIC | 0.782 | 0.705 | 0.019 | 0.615 | 0.76 | IVW |
| 5 | G-CSF | 0.949 | 0.922 | 0.017 | 0.651 | 0.92 | IVW |
| 6 | GROA | 0.523 | 0.771 | -0.080 | 0.198 | 0.52 | IVW |
| 7 | HGF | 0.381 | 0.260 | 0.004 | 0.922 | 0.44 | IVW |
| 8 | IFN-G | 0.333 | 0.270 | -0.027 | 0.545 | 0.39 | IVW |
| 9 | IL-1B | 0.991 | 0.990 | 0.026 | 0.655 | 1 | IVW |
| 10 | IL-1RA | 0.752 | 0.616 | 0.001 | 0.990 | 0.81 | IVW |
| 11 | IL-2 | 0.663 | 0.522 | -0.008 | 0.887 | 0.74 | IVW |
| 12 | IL-2RA | 0.111 | 0.062 | 0.002 | 0.984 | 0.15 | IVW |
| 13 | IL-4 | 0.720 | 0.581 | -0.003 | 0.933 | 0.68 | IVW |
| 14 | IL-5 | 0.133 | 0.095 | -0.039 | 0.628 | 0.2 | IVW |
| 15 | IL-6 | 0.569 | 0.458 | -0.017 | 0.654 | 0.58 | IVW |
| 16 | IL-7 | 0.609 | 0.464 | -0.002 | 0.973 | 0.68 | IVW |
| 17 | IL-8 | 0.957 | 0.952 | 0.032 | 0.574 | 0.92 | IVW |
| 18 | IL-9 | 0.882 | 0.798 | -0.016 | 0.775 | 0.9 | IVW |
| 19 | IL-10 | 0.532 | 0.766 | -0.054 | 0.205 | 0.44 | IVW |
| 20 | IL-12-P70 | 0.493 | 0.433 | -0.026 | 0.482 | 0.51 | IVW |
| 21 | IL-13 | 0.274 | 0.189 | -0.024 | 0.728 | 0.32 | IVW |
| 22 | IL-16 | 0.786 | 0.678 | -0.018 | 0.742 | 0.8 | IVW |
| 23 | IL-17 | 0.850 | 0.796 | -0.077 | 0.701 | / | IVW |
| 24 | IL-18 | 0.259 | 0.213 | 0.043 | 0.528 | 0.34 | IVW |
| 25 | IP-10 | 0.623 | 0.601 | -0.044 | 0.434 | 0.64 | IVW |
| 26 | M-CSF | 0.330 | 0.234 | -0.027 | 0.725 | 0.41 | IVW |
| 27 | MCP-1-MCAF | 0.219 | 0.151 | -0.018 | 0.703 | 0.31 | IVW |
| 28 | MCP-3 | 0.481 | 0.344 | -0.008 | 0.942 | 0.56 | IVW |
| 29 | MIF | 0.285 | 0.193 | 0.020 | 0.776 | 0.38 | IVW |
| 30 | MIG | 0.994 | 0.994 | 0.023 | 0.669 | 1 | IVW |
| 31 | MIP-1A | 0.880 | 0.785 | -0.010 | 0.862 | 0.95 | IVW |
| 32 | MIP-1B | 0.539 | 0.596 | 0.039 | 0.318 | 0.51 | IVW |
| 33 | PDGF-BB | 0.682 | 0.971 | 0.055 | 0.183 | 0.58 | IVW |
| 34 | RANTES | 0.269 | 0.171 | 0.003 | 0.962 | 0.37 | IVW |
| 35 | SCF | 0.288 | 0.219 | 0.023 | 0.609 | 0.26 | IVW |
| 36 | SCGF-B | 0.796 | 0.926 | 0.062 | 0.291 | 0.76 | IVW |
| 37 | SDF-1A | 0.403 | 0.280 | -0.007 | 0.874 | 0.45 | IVW |
| 38 | TNF-A | 0.662 | 0.518 | -0.002 | 0.967 | 0.7 | IVW |
| 39 | TNF-B | 0.947 | 0.926 | -0.041 | 0.622 | 0.97 | IVW |
| 40 | TRAIL | 0.659 | 0.535 | 0.012 | 0.734 | 0.66 | IVW |
| 41 | VEGF | 0.249 | 0.267 | -0.044 | 0.354 | 0.26 | IVW |

**Table S6. Details of KOA predicting SNPs with inflammatory cytokines**

| **SNP** | **effect allele** | **other allele** | **chrom** | **se** | **pval** | **EAF** | **Beta** | ***F*** |
| --- | --- | --- | --- | --- | --- | --- | --- | --- |
| rs1078301 | T | A | 9 | 0.011 | 1.269E-10 | 0.269 | 0.068 | 41.032 |
| rs12470967 | G | A | 2 | 0.010 | 1.49799E-08 | 0.575 | -0.058 | 32.148 |
| rs143384 | G | A | 20 | 0.010 | 4.77309E-23 | 0.403 | -0.094 | 96.867 |
| rs4775006 | A | C | 15 | 0.009 | 8.40001E-10 | 0.411 | 0.058 | 37.809 |
| rs56116847 | A | G | 12 | 0.010 | 3.19396E-10 | 0.356 | 0.061 | 39.807 |
| rs8067763 | A | G | 17 | 0.010 | 2.386E-09 | 0.594 | -0.057 | 35.496 |

Abbreviations: EAF: effect allele frequency, Beta: effect size of exposure on outcome, se: standard error; chrom: chromosome
