# Supplementary material for: Exploring causal correlations between inflammatory cytokines and knee osteoarthritis: a two-sample Mendelian randomization
Source: Front Immunol. 2024 Apr 18;15:1362012. doi: 10.3389/fimmu.2024.1362012 (PMC11063282; doi:10.3389/fimmu.2024.1362012)
Supplement: Supplementary file 2 [file Table_2.pdf]

STROBE-MR checklist of recommended items to address in reports of Mendelian randomization studies[1 2]

Item No.	Section	Checklist item	Page No.	Relevant text from manuscript
1	**TITLE and ABSTRACT**	Indicate Mendelian randomization (MR) as the study's design in the title and/or the abstract if that is a main purpose of the study	1	Exploring causal correlations between inflammatory cytokines and knee osteoarthritis: A two-sample Mendelian randomization
	**INTRODUCTION**			
2	**Background**	Explain the scientific background and rationale for the reported study. What is the exposure? Is a potential causal relationship between exposure and outcome plausible? Justify why MR is a helpful method to address the study question	2-3	The association between inflammation and KOA has been demonstrated in animal model studies. Furthermore, certain inflammatory cytokines, such as IL-1 and tumor necrosis factor (TNF-α), can be detected during early lesions of KOA, a finding that suggests that inflammatory cytokines are associated with KOA. The expression levels of inflammatory cytokines in joint fluids influence KOA to some extent. However, relevant studies have mainly focused on the effects of IL-1 and TNF-a on KOA, and few studies regarding the association between other inflammatory cytokines and KOA exist. In addition, owing to the limitations of the traditional study design and the inability of the current study to completely exclude confounding factors, the causal relationship between inflammatory cytokines and KOA remains unclear and should be confirmed by additional studies. Mendelian randomization (MR) uses genetic variants associated with modifiable exposures (or risk factors) to assess their possible causal relationship with outcomes. Single nucleotide polymorphisms (SNPs) are commonly used as IVs to assess potential causal associations with outcomes. Because genetic variants are randomly assigned at the time of conception, potential sources of error are eliminated, and a clear causal chain is established. This minimizes potential bias due to confounding factors and reverse causation, thus increasing the reliability of the results. Current MR in KOA mostly focuses on MR between two diseases or influencing factors, such as OA with cardiovascular disease and osteoporosis. However, there is a notable gap between the studies addressing the potential causal

				relationship between KOA and inflammatory cytokine levels. To address this gap, we aimed to assess the potential causal association between inflammatory cytokines and KOA using two-sample bidirectional MR analysis
3	**Objectives**	State specific objectives clearly, including pre-specified causal hypotheses (if any). State that MR is a method that, under specific assumptions, intends to estimate causal effects	3	To address this gap, we aimed to assess the potential causal association between inflammatory cytokines and KOA using two-sample bidirectional MR analysis. We first extracted validated genetic IVs for 41 inflammatory cytokines from the genome-wide association study (GWAS) data and analyzed their association with KOA. The direction of causality was further explored using reverse exposure and outcomes. Our study findings potentially provide new strategies for KOA prevention.
	**METHODS**			
4	**Study design and data sources**	Present key elements of the study design early in the article. Consider including a table listing sources of data for all phases of the study. For each data source contributing to the analysis, describe the following:		
	a)	Setting: Describe the study design and the underlying population, if possible. Describe the setting, locations, and relevant dates, including periods of recruitment, exposure, follow-up, and data collection, when available.	3	The KOA data for this study were obtained from the UK Biobank and Arthritis Research UK Osteoarthritis Genetics (arcOGEN). This study included 24,955 patients and 378,169 controls of European ancestry (https://was.mrcieu.ac.uk/datasets/ebi-a-GCST007090/). The UK Biobank recruited participants aged 40–69 between 2006–2010. ArcOGEN is a collection of patients of European ancestry with KOA. For 41 inflammatory cytokines, data were obtained from a meta-analysis combining the results of the Finnish Young Adult Cardiovascular Risk Study (YFS) and the FINRISK survey between 1980–2011, which included 8,293 Finnish individuals (https://data.bris.ac.uk/data/dataset/3g3i5smgghp0s2uvm1doflkx9x). The mean age of the participants in the YFS an FINRISK studies were 37 and 60 years, respectively.
	b)	Participants: Give the eligibility criteria, and the sources and methods of selection of participants. Report the sample size, and whether any power or sample size calculations were carried out prior to the main analysis	3	The KOA data for this study were obtained from the UK Biobank and Arthritis Research UK Osteoarthritis Genetics (arcOGEN). This study included 24,955 patients and 378,169 controls of European ancestry. For 41 inflammatory cytokines, data were obtained from a meta-

					analysis combining the results of the Finnish Young Adult Cardiovascular Risk Study (YFS) and the FINRISK survey between 1980–2011, which included 8,293 Finnish individuals.
		c)	Describe measurement, quality control and selection of genetic variants	4	First, we set P<5×10-8 as the genome-wide significance threshold to select SNPs that were strongly associated with KOA and inflammatory cytokines. Because very few SNPs were identified for some of the cytokines upon exposure, a higher cutoff value (P<5×10-6) to ensure that there were enough SNPs for further MR analysis [40, 41]. Secondly, to avoid linkage disequilibrium, instrumental variables were removed to ensure mutual independence of these instrumental variables (r2=0.001, kb=10,000).
		d)	For each exposure, outcome, and other relevant variables, describe methods of assessment and diagnostic criteria for diseases	3	Diagnosis of KOA was based on clinical signs of the disease and the need for arthroplasty or imaging evidence (Kelgren-Lawrence≥2) to determine the diagnosis.
		e)	Provide details of ethics committee approval and participant informed consent, if relevant	No	Ethical approval was not required because the analyses involved public abstract-level datasets.
5	**Assumptions**		Explicitly state the three core IV assumptions for the main analysis (relevance, independence and exclusion restriction) as well assumptions for any additional or sensitivity analysis	3/4	MR was used to analyze the relationship between inflammatory cytokines and KOA. Figure 1 shows the two-sample bidirectional MR study design. There are three core assumptions of MR analysis: relevance, independence, and exclusion. (1) Relevance: the variables selected as genetic instruments are closely associated with exposures; (2) independence: genetic variation is not associated with confounding factors; and (3) exclusion restrictions: genetic variation affects outcomes only through exposures instead of other pathways. The IVW method served as the primary approach for the MR analysis, and Cochran's Q value was computed for both the IVW and MR-Egger estimates to gauge heterogeneity. Horizontal pleiotropy was evaluated using the MR-Egger intercept test [42] and anomalous SNPs were identified using MR-PRESSO [43]. The stability of the results was examined using the leave-one-out method.

6	**Statistical methods: main analysis**		Describe statistical methods and statistics used		
		a)	Describe how quantitative variables were handled in the analyses (i.e., scale, units, model)	No	
		b)	Describe how genetic variants were handled in the analyses and, if applicable, how their weights were selected	4	First, we set P<5×10-8 as the genome-wide significance threshold to select SNPs that were strongly associated with KOA and inflammatory cytokines. Because very few SNPs were identified for some of the cytokines upon exposure, a higher cutoff value (P<5×10-6) to ensure that there were enough SNPs for further MR analysis [40, 41]. Secondly, to avoid linkage disequilibrium, instrumental variables were removed to ensure mutual independence of these instrumental variables (r2=0.001, kb=10,000). Third, the F statistic was used to determine the IV exposure correlations. The F value of the SNP was calculated to determine the presence of a weak IV bias. If F>10, the correlation is considered strong enough to avoid weak IV bias.
		c)	Describe the MR estimator (e.g. two-stage least squares, Wald ratio) and related statistics. Detail the included covariates and, in case of two-sample MR, whether the same covariate set was used for adjustment in the two samples	3	The two datasets used in the MR analysis were derived from publicly available GWAS summary data. The KOA data for this study were obtained from the UK Biobank and Arthritis Research UK Osteoarthritis Genetics (arcOGEN). This study included 24,955 patients and 378,169 controls of European ancestry [38] (https://was.mrcieu.ac.uk/datasets/ebi-a-GCST007090/). The UK Biobank recruited participants aged 40–69 between 2006–2010. ArcOGEN is a collection of patients of European ancestry with KOA. For 41 inflammatory cytokines, data were obtained from a meta-analysis combining the results of the Finnish Young Adult Cardiovascular Risk Study (YFS) and the FINRISK survey between 1980–2011, which included 8,293 Finnish individuals [39] (https://data.bris.ac.uk/data/dataset/3g3i5smgghp0s2uvm1doflkx9x). The mean age of the participants in the YFS an FINRISK studies were 37 and 60 years, respectively.
		d)	Explain how missing data were addressed	No	
		e)	If applicable, indicate how multiple testing was addressed	No	

7	**Assessment of assumptions**	Describe any methods or prior knowledge used to assess the assumptions or justify their validity	4	The F value of the SNP was calculated to determine the presence of a weak IV bias. If F>10, the correlation is considered strong enough to avoid weak IV bias. Causality assessments were conducted using diverse methods including inverse variance weighting (IVW), MR-Egger, weighted median, weighted modal, and simple modal analyses.
8	**Sensitivity analyses and additional analyses**	Describe any sensitivity analyses or additional analyses performed (e.g. comparison of effect estimates from different approaches, independent replication, bias analytic techniques, validation of instruments, simulations)	4	The IVW method served as the primary approach for the MR analysis, and Cochran's Q value was computed for both the IVW and MR-Egger estimates to gauge heterogeneity. Horizontal pleiotropy was evaluated using the MR-Egger intercept test [42] and anomalous SNPs were identified using MR-PRESSO [43]. The stability of the results was examined using the leave-one-out method, wherein each SNP was systematically excluded to assess its impact on supporting a causal association [44]. In cases where IVW analysis yielded a significant result (P<0.05) and no evidence of horizontal pleiotropy or heterogeneity was found, the result could be considered positive, even if other methods did not yield a significant result, as long as the direction of the beta remained consistent [42]. In cases where horizontal pleiotropy was present but no heterogeneity was detected, the MR-Egger method was used. In cases where heterogeneity was observed, but multidirectionality was not present, the analysis was performed using the IVW method.
9	**Software and pre-registration**			
	a)	Name statistical software and package(s), including version and settings used	4	In this study, we explored the potential causal relationship between inflammatory cytokines and KOA using inflammatory cytokines and KOA as exposure and outcome variables, respectively. Analysis was performed using R4.3.2 software and the R package "Two sample MR".
	b)	State whether the study protocol and details were pre-registered (as well as when and where)	No	unregistered
	**RESULTS**			
10	**Descriptive data**			

		a)	Report the numbers of individuals at each stage of included studies and reasons for exclusion. Consider use of a flow diagram	3	The KOA data for this study were obtained from the UK Biobank and Arthritis Research UK Osteoarthritis Genetics (arcOGEN). This study included 24,955 patients and 378,169 controls of European ancestry [38] (https://was.mrcieu.ac.uk/datasets/ebi-a-GCST007090/). For 41 inflammatory cytokines, data were obtained from a meta-analysis combining the results of the Finnish Young Adult Cardiovascular Risk Study (YFS) and the FINRISK survey between 1980–2011, which included 8,293 Finnish individuals [39] (https://data.bris.ac.uk/data/dataset/3g3i5smgghp0s2uvm1doflkx9x). The mean age of the participants in the YFS an FINRISK studies were 37 and 60 years, respectively.
		b)	Report summary statistics for phenotypic exposure(s), outcome(s), and other relevant variables (e.g. means, SDs, proportions)	4-7	Details see Table
		c)	If the data sources include meta-analyses of previous studies, provide the assessments of heterogeneity across these studies	No	
		d)	For two-sample MR:<br>i. Provide justification of the similarity of the genetic variant-exposure associations between the exposure and outcome samples<br>ii. Provide information on the number of individuals who overlap between the exposure and outcome studies	3	The populations in both samples were from Europe, so there was little ethnic heterogeneity.
11	**Main results**				
		a)	Report the associations between genetic variant and exposure, and between genetic variant and outcome, preferably on an interpretable scale	4-7	Details see Table1-2
		b)	Report MR estimates of the relationship between exposure and outcome, and the measures of uncertainty from the MR analysis, on an interpretable scale, such as odds ratio or relative risk per SD difference	4-7	Details see Table1-2
		c)	If relevant, consider translating estimates of relative risk into absolute risk for a meaningful time period	No	
		d)	Consider plots to visualize results (e.g. forest plot, scatterplot of associations between genetic variants and outcome versus between genetic variants and exposure)	8	See Figure 2
12	**Assessment of assumptions**				

		a)	Report the assessment of the validity of the assumptions	See supplement file	The supplement is provided. （Table S2,5）
		b)	Report any additional statistics (e.g., assessments of heterogeneity across genetic variants, such as I^2, Q statistic or E-value)	See supplement file	The supplement is provided. （Table S2,5）
13	**Sensitivity analyses and additional analyses**				
		a)	Report any sensitivity analyses to assess the robustness of the main results to violations of the assumptions	5-7	The main results of the MR analysis using 41 inflammatory cytokines as exposures and KOA as the outcome are shown in Tables 1 and S1. The IVW results showed that genetically predicted elevated levels of Granulocyte colony-stimulating factor (G-CSF) were negatively correlated with the risk of developing KOA (OR:0.93, 95%CI:0.89–0.99, P=0.015). There was no evidence of potential horizontal pleiotropy (P=0.173) or heterogeneity (P=0.597) (Table S2). MR-PRESSO analyses did not identify any SNP abnormalities in the G-CSF. The IVW results showed that genetically predicted increases in KOA occurrence were negatively associated with MIP-1A levels (odds ratio [OR], 0.72; 95%CI:0.54–0.97, P=0.032). MR-Egger analysis did not reveal any potential horizontal pleiotropy (P=0.862) or heterogeneity (P=0.880) of SNPs for exposure factors. MR-PRESSO analyses did not reveal outliers in the SNPs for the exposure factors (Table S5).
		b)	Report results from other sensitivity analyses or additional analyses	5-7	The leave-one-out method demonstrates the stability of the results (Fig. S1). Leave-one-out analysis demonstrated the stability of the results (Fig S2).
		c)	Report any assessment of direction of causal relationship (e.g., bidirectional MR)	4	To ensure an adequate number of SNPs for further MR analysis, a significance threshold of P<5×10-6 was selected when screening for SNPs related to each inflammatory cytokine and KOA. The F-statistics for the SNPs of the 41 inflammatory cytokines were 20.77–782.26. The F-statistic for all instrumental variables was >10,

					indicating no weak instrumental variable bias (Tables S1-S3).<br>To determine the primary analytical tool, hypothesis checks were performed for all 41 inflammatory cytokines. The IVW method was used as the primary analytical method for all cytokines, except IL-1RA, for which there was no evidence of heterogeneity or weak interference. For IL-1RA, MR-Egger was chosen as the primary method and the reason for this was because the P-values of the Q-tests for both IVW and MR-Egger were <0.05 (P=0.032, P=0.040).
		d)	When relevant, report and compare with estimates from non-MR analyses	No	
		e)	Consider additional plots to visualize results (e.g., leave-one-out analyses)	See supplement file	The supplement is provided. (figS1-2)
	**DISCUSSION**				
14	**Key results**		Summarize key results with reference to study objectives	8	This study used a two-sample MR analysis to explore the potential causal relationship between inflammatory cytokines and KOA. First, we identified causal relationships between 41 inflammatory cytokines and KOA and found that lower levels of G-CSF were associated with a high risk of developing KOA, suggesting that G-CSF may be a potential upstream cause of KOA. In addition, when KOA is used as an exposure variable in MR, it may lead to lower MIP-1A levels via pathogenic pathways; therefore, MIP-1A may be located downstream of disease progression. In summary, our analysis suggests that G-CSF is involved in KOA pathogenesis. MIP-1A has a protective effect against KOA, as evidenced by the reduction in MIP-1A levels when KOA was considered an exposure factor.
15	**Limitations**		Discuss limitations of the study, taking into account the validity of the IV assumptions, other sources of potential bias, and imprecision. Discuss both direction and magnitude of any potential bias and any efforts to address them	10	This study has several limitations. First, our survey data were derived from two large-scale GWAS datasets, which make this study less statistically valid. However, owing to the lack of data, we were unable to perform subgroup analyses of the variables to refine the results of this study. Therefore, they must be validated in future large-sample clinical trials. Second, the GWAS data used in this study were derived from individuals of European origin, and there may be racial bias. Thus, the generalizability of our findings to other

				populations should be validated using local data. Third, a significance threshold with a P<5×10-6 was used in the exposed GWAS data because the number of genome-wide significant SNP at a threshold of P<5×10-8 was too small to support this study. Fourth, the scope of this study was limited to examining the correlation between the 41 inflammatory cytokines and KOA. Given the diversity of inflammatory cytokines, future studies should be extended to the latest 91 inflammatory cytokines to deepen our understanding of the causal relationships between inflammatory cytokines and KOA.
16	**Interpretation**			
		a) Meaning: Give a cautious overall interpretation of results in the context of their limitations and in comparison with other studies	9-10	The previous studies investigated the potential causal relationship between cytokines and OA, suggesting a correlation between CX3CL1, MCP4, and CCL25 and OA [78]. Another study noted a causal relationship between the inflammatory cytokines MCSF and vascular endothelial growth factor (VEGF) and OA [79], but these results were not validated in our analysis. Potential explanations include differences in study focus, with previous research encompassing various types of OA, while our study specifically examined KOA. Variation in the selection of inflammatory cytokines across studies, each with distinct biological functions and expression patterns, may also contribute to differing results. Additionally, the heterogeneity of study populations could impact cytokine expression levels and patterns, influencing study outcomes. Notably, our study had a large sample size and assessed numerous cytokines. Furthermore, we identified previously unexplored causal relationships, such as the association between G-CSF and MIP-1A with KOA. These novel findings warrant further validation in future research to ascertain the potential of G-CSF and MIP-1A as biomarkers for KOA prevention and treatment.
		b) Mechanism: Discuss underlying biological mechanisms that could drive a potential causal relationship between the investigated exposure and the outcome, and whether the gene-environment equivalence assumption is reasonable. Use causal language carefully, clarifying that IV estimates may provide causal effects only under certain assumptions	8-9	Our findings suggested that reduced G-CSF levels were associated with an increased risk of KOA. G-CSF (also known as CSF-3) is a hematopoietic growth factor produced primarily by macrophages, vascular endothelial cells, fibroblasts, and bone marrow mesenchymal stromal cells [45]. G-CSF is widely found in the body and the circulatory

system [46] and is involved in chronic inflammatory autoimmune diseases such as rheumatoid arthritis (RA) [47]. RA contributes directly to joint deterioration by mediating the release of a variety of cells, including macrophages and lymphocytes, as well as inflammatory factors: TNF-α, IL-1 and matrix metalloproteinases [48], which in turn manifests as joint deformities and dysfunction. This finding implies that G-CSF may have similar effects in patients with KOA. G-CSF primarily acts on hematopoietic progenitor cells to promote proliferation and differentiation, and stimulates granulocyte maturation and release [49-51]. Reduced levels of G-CSF lead to decreased chemotaxis of granulocytes to damaged tissues [52], leading to decreased granulocyte numbers and activity [53, 54], thereby affecting immune cell function [55] and leading to reduced repair capacity of tissues, such as articular cartilage. Damage to articular cartilage and subchondral bone is an underlying pathological process of OA [56]. G-CSF affects the survival and function of chondrocytes in the joint fluid [57], and reduced levels alter the state of these cells, affecting the stability of joint tissues [58], ultimately leading to KOA. MIP-1A (also known as CCL3), belongs to the chemokine CC subfamily. MIP-1A is produced by a variety of cell types, including monocytes, fibroblasts, vascular endothelial cells, and smooth muscle cells [59], has chemotactic effects on monocytes, T cells, B cells [60,61], and is involved in the pathogenesis of several inflammatory diseases such as RA and asthma [62-64]. In patients with RA, MIP-1A triggers signaling pathways by binding to receptors on the surface of chondrocytes, leading to altered activation of intracellular molecules and inhibition of proteoglycan synthesis, ultimately affecting chondrocyte function [65,66]. In patients with RA, decreased MIP-1A expression correlates with disease severity, and there is a trend toward decreased MIP-1A expression with decreased macrophage infiltration [67]. RA and KOA are both inflammatory diseases [68]; therefore, it is hypothesized that MIP-1A may have a similar effect on KOA. A study on the synovial fluid also reported that MIP-1A levels decreased with OA

			progression [69]. Lowering MIP-1A levels may exert a protective effect against KOA through multiple pathways, including influencing immune cell activity, slowing inflammatory responses, and maintaining articular cartilage stability. First, lowering MIP-1A levels decreases immune cell activity [70]. MIP-1A reduces the destructive effects of macrophages on joint tissues by attenuating their activation, thereby protecting joint structures, maintaining joint tissues [71], and slowing the progression of arthritis. Secondly, as an important chemokine [72], reduced levels of MIP-1A may lead to reduced immune cell chemotaxis in the affected joint region [73]. This in turn slows the inflammatory response [74], thereby reducing inflammation and attenuating joint tissue degradation. Finally, MIP-1A affects chondrocyte function [75], leading to limited cellular synthesis of proteoglycans, which reduces proteoglycan synthesis and exacerbates articular cartilage damage [76]. Therefore, a reduction in MIP-1A levels promotes proteoglycan synthesis, which reduces the risk of articular cartilage damage and protects joints. Here, we found that MIP-1A levels were decreased in patients with KOA, whereas the study by Guo et al. [77] reported that MIP-1A levels were increased in patients with KOA, leading to a discrepancy, which may be due to the difference in the sample sizes of the studies, as well as the racial differences between the European and Asian populations. Therefore, future studies should explore the potential mechanism of the negative correlation between KOA and MIP-1A to reveal the specific role of MIP-1A in the pathogenesis of KOA, which will provide new perspectives on the treatment strategy of KOA and provides new ideas for discovering new ways to treat KOA.
c)	Clinical relevance: Discuss whether the results have clinical or public policy relevance, and to what extent they inform effect sizes of possible interventions	10	The previous studies investigated the potential causal relationship between cytokines and OA, suggesting a correlation between CX3CL1, MCP4, and CCL25 and OA [78]. Another study noted a causal relationship between the inflammatory cytokines MCSF and vascular endothelial growth factor (VEGF) and OA [79], but these results were not validated in our analysis. Potential explanations include differences in study focus, with previous research encompassing various

				types of OA, while our study specifically examined KOA. Variation in the selection of inflammatory cytokines across studies, each with distinct biological functions and expression patterns, may also contribute to differing results. Additionally, the heterogeneity of study populations could impact cytokine expression levels and patterns, influencing study outcomes. Notably, our study had a large sample size and assessed numerous cytokines. Furthermore, we identified previously unexplored causal relationships, such as the association between G-CSF and MIP-1A with KOA. These novel findings warrant further validation in future research to ascertain the potential of G-CSF and MIP-1A as biomarkers for KOA prevention and treatment.
17	**Generalizability**	Discuss the generalizability of the study results (a) to other populations, (b) across other exposure periods/timings, and (c) across other levels of exposure	10	This study has several limitations. First, our survey data were derived from two large-scale GWAS datasets, which make this study less statistically valid. However, owing to the lack of data, we were unable to perform subgroup analyses of the variables to refine the results of this study. Therefore, they must be validated in future large-sample clinical trials.
	**OTHER INFORMATION**			
18	**Funding**	Describe sources of funding and the role of funders in the present study and, if applicable, sources of funding for the databases and original study or studies on which the present study is based	10	The authors received no specific funding for this work.
19	**Data and data sharing**	Provide the data used to perform all analyses or report where and how the data can be accessed, and reference these sources in the article. Provide the statistical code needed to reproduce the results in the article, or report whether the code is publicly accessible and if so, where	10	We thank all participants who contributed to the GWAS data used in this study.(https://was.mrcieu.ac.uk/datasets/ebi-a-GCST007090/). (https://data.bris.ac.uk/data/dataset/ 3g3i5smgghp0s2uvm1doflkx9x).
20	**Conflicts of Interest**	All authors should declare all potential conflicts of interest	10	The authors declare that the research was conducted in the absence of any commercial or financial relationships that could be construed as a potential conflict of interest.

This checklist is copyrighted by the Equator Network under the Creative Commons Attribution 3.0 Unported (CC BY 3.0) license.

1. Skrivankova VW, Richmond RC, Woolf BAR, Yarmolinsky J, Davies NM, Swanson SA, et al. Strengthening the Reporting of Observational Studies in Epidemiology

using Mendelian Randomization (STROBE-MR) Statement. JAMA. 2021;under review.

2. Skrivankova VW, Richmond RC, Woolf BAR, Davies NM, Swanson SA, VanderWeele TJ, et al. Strengthening the Reporting of Observational Studies in Epidemiology using Mendelian Randomisation (STROBE-MR): Explanation and Elaboration. BMJ. 2021;375:n2233.
